# AdaSwitch3D: Reliability-Aware Human-in-the-Loop Artificial Intelligence for Breast Tumor Segmentation in Dynamic Contrast-Enhanced MRI Across Unseen Centers

**DOI:** 10.3390/diagnostics16203378

**Published:** 2026-10-17

**Authors:** Easa Alalwany, Bader Alsharif

**Affiliations:** 1 Department of Computer Science, College of Computer Science and Engineering, Taibah University, Yanbu 46421, Saudi Arabia; 2 Health and Life Research Center, Taibah University, Madinah 42353, Saudi Arabia; 3 Department of Electrical Engineering and Computer Science, Florida Atlantic University, Boca Raton, FL 33431, USA; balsharif2020@fau.edu

**Keywords:** breast cancer, DCE-MRI, tumor segmentation, domain adaptation, few-shot learning, teacher–student self-training, case-wise routing, review prioritization, cross-center robustness, multi-seed evaluation

## Abstract

**Background/Objectives:** Deep learning models for breast tumor segmentation in dynamic contrast-enhanced magnetic resonance imaging (DCE-MRI) often lose accuracy at centers that did not contribute training data. We investigated whether case-wise routing between two low-resource adapted models, combined with an uncertainty-based review-prioritization signal, behaves consistently under severe cross-center domain shift. **Methods:** Using the four public collections of the MAMA-MIA dataset (DUKE, ISPY1, ISPY2, NACT; 1231 cases), we performed four-fold leave-one-center-out experiments in which each held-out center contributed only five labeled cases and a pool of unlabeled scans. We compared source-only transfer, five-shot fine-tuning, and teacher–student self-training with AdaSwitch3D, which routes each case between the two adapted branches using 17 label-free features and produces a heuristic review-prioritization signal. The complete pipeline—data splits, five-shot selection, training, adaptation, router construction, and referral calibration—was repeated with five random seeds, and each seed was evaluated once on 80 untouched test cases (400 seed-specific test observations). **Results:** Across five independent repetitions, mean Dice was 0.325 ± 0.051 for source-only transfer, 0.353 ± 0.052 for five-shot fine-tuning, 0.372 ± 0.047 for teacher–student adaptation, and 0.377 ± 0.057 for AdaSwitch3D; a validation-selected per-center strategy reached 0.376 ± 0.044. AdaSwitch3D exceeded five-shot fine-tuning in all five seeds and teacher–student adaptation in four, but the difference between AdaSwitch3D and teacher–student adaptation was small (mean +0.005) and varied by seed, while inference required both branches (approximately twice the single-model time). Observed test referral rates ranged from 23.8% to 56.3% against a 20% validation target, and low-Dice (<0.20) failure capture ranged from 21.7% to 71.8%. In the prespecified case-level analysis of seed 42, AdaSwitch3D improved on source-only transfer and five-shot fine-tuning (Holm-adjusted p=0.021 for each), whereas the comparison with teacher–student adaptation did not provide evidence of a difference (Holm-adjusted p=0.508). **Conclusions:** Absolute segmentation accuracy remained low, the incremental benefit of case-wise routing over teacher–student adaptation was small and seed-dependent, and referral behavior was unstable across repetitions. The principal finding is therefore that cross-center adaptation, case-wise model selection, and heuristic referral remain sensitive under limited target supervision. AdaSwitch3D should be interpreted as an exploratory case study of these challenges rather than as a clinically validated segmentation or referral system.

## 1. Introduction

Breast cancer is the most frequently diagnosed cancer in women and a leading cause of cancer-related death worldwide [1]. DCE-MRI is a central modality for high-risk screening, local staging, treatment planning, and the assessment of response to neoadjuvant chemotherapy, owing to its high sensitivity for invasive disease [2]. Accurate tumor delineation on DCE-MRI supports volumetric burden estimation, response assessment, radiomic characterization, and treatment planning, but manual segmentation of volumetric multi-phase data is time-consuming and subject to appreciable inter- and intra-reader variability [3]. Automated breast tumor segmentation on DCE-MRI is challenging because lesions are small relative to the imaged volume and because background parenchymal enhancement competes with tumor signal [4].

Methodologically, medical image segmentation has been dominated by convolutional encoder–decoder networks, in particular the U-Net family [5,6,7]. Transformer-based architectures, which model long-range context through self-attention, have since been adapted to volumetric segmentation as pure or hybrid encoders [8,9] and have been surveyed extensively across medical imaging tasks [10]. Reviews and benchmarks that compare convolutional neural network (CNN) and transformer architectures, including for brain tumor classification in MRI [11], document this architectural evolution. Related computer-vision studies outside medical imaging have likewise compared CNN/ViT families and newer state-space vision models under application-specific accuracy and complexity constraints [12,13]. These cross-domain results are not evidence for breast MRI performance, but they reinforce that architecture rankings are task-dependent. Brain tumor classification is an image-level task that differs from voxel-level breast tumor segmentation in DCE-MRI, so conclusions about the relative ranking of architectures do not transfer directly to our setting. A point that does carry over is that architectural gains reported under matched training and test distributions do not by themselves establish robustness at new institutions. Careful re-evaluations have also shown that well-configured CNN-based U-Nets remain highly competitive with newer designs [14]. For these reasons, the present study uses a single, standard 3D U-Net backbone and focuses on adaptation, routing, and referral behavior rather than on architecture.

In deployment, accuracy at one institution frequently does not transfer to another. Differences in field strength, scanner vendor, coil configuration, contrast agent dose and timing, temporal resolution of the dynamic series, fat-suppression technique, and postprocessing combine to produce a distribution shift between the data a model was trained on and the data it meets at deployment [15,16]. Performance losses at external sites have been documented for deep learning models in radiology [17] and for segmentation networks in particular [18,19,20]. For breast DCE-MRI specifically, a balanced leave-one-center-out analysis on the same four MAMA-MIA centers quantified this degradation directly and reported substantial center-to-center variation [21].

The constraints most centers face are asymmetric. Manual annotation of volumetric breast DCE-MRI is expensive and scarce, whereas unlabeled clinical examinations accumulate during routine care [20]. A realistic adaptation workflow should therefore assume only a small labeled subset at the target center, exploit a larger unlabeled target pool, use a standard backbone rather than a bespoke architecture, and expose a reliability signal so that uncertain cases can be prioritized for human review rather than accepted silently [22,23,24]. Prior domain adaptation work for medical segmentation spans feature alignment [25], image translation [26], and self-training or pseudo-labeling [27,28,29], and few-shot transfer learning adapts a source model using a handful of target labels [30,31].

Reliability mechanisms are a second, largely separate line of work. Selective prediction and uncertainty estimation provide principled mechanisms for abstention [23,32,33], but segmentation networks are frequently miscalibrated [34], and dedicated quality-control models are typically needed to predict segmentation failure on a per-case basis [35]. Human-in-the-loop approaches keep clinicians involved in model development and deployment [36], and learning-to-defer methods train a rejector that decides when a model should hand a case to an expert [37,38]. Conformal prediction offers distribution-free coverage guarantees under exchangeability [39], an assumption that cross-center shift explicitly violates. How adaptation, case-level model selection, and review prioritization behave together, under a leakage-controlled cross-center protocol and across repeated randomizations, has received comparatively little attention.

The goal of this study is therefore methodological: to characterize the performance, variability, and limitations of low-resource adaptation, case-wise model routing, and heuristic referral under severe cross-center domain shift. AdaSwitch3D is evaluated as an exploratory instantiation of case-wise routing and review prioritization rather than as a clinically validated or preferred deployment architecture.

We use a strict leave-one-center-out design as a cross-center stress test to study how case-wise routing between two adapted segmenters, and an uncertainty-based referral signal derived from them, behave under severe domain shift with minimal target supervision. The study does not attempt to establish a clinically acceptable segmentation system: absolute Dice values remained modest under every strategy, no clinically validated minimum accuracy for any intended use was defined in advance, and workflow utility could only be established through reader studies and task-specific performance criteria, which were not part of this work. Because results from a single randomization can be unstable in small, heterogeneous cohorts [40,41], the complete pipeline was repeated with five random seeds, and we report the resulting variability, including unfavorable seeds, as a primary result.

The individual components we use, including fine-tuning, teacher–student self-training, logistic-regression model selection, and threshold-based referral, are established techniques, and we do not claim a new learning algorithm. Our contribution is a system-level methodological integration and a rigorous evaluation of that integration under cross-center domain shift:

Our contributions are as follows:

1. A five-seed evaluation of cross-center adaptation that quantifies sensitivity to data partitioning, five-shot case selection, training, routing, and referral calibration.
2. A controlled comparison of source-only transfer, five-shot fine-tuning, and teacher–student adaptation using the same 3D U-Net backbone under a strict leave-one-center-out protocol.
3. A characterization of the performance and computational trade-offs of validation-selected center-level and case-wise model selection.
4. AdaSwitch3D as an exploratory implementation of label-free case-wise routing between two adapted segmentation branches, used as an experimental testbed rather than proposed as a preferred deployment method.
5. A negative-result analysis of heuristic review prioritization, demonstrating unstable workload–failure-capture behavior and characterizing the mechanisms underlying missed low-Dice failures.
6. An analysis of the limitations that must be addressed by future quality-estimation, learning-to-defer, or risk-controlling approaches before clinical deployment can be considered.

The remainder of the paper is organized as follows. Section 2 reviews related work; Section 3 describes the dataset, models, AdaSwitch3D, the multi-seed design, and the evaluation; Section 4 presents the five-seed results followed by the detailed seed-42 analysis; and Section 5, Section 6 and Section 7 discuss implications, limitations, and conclusions.

## 2. Related Work

### 2.1. Breast DCE-MRI Tumor Segmentation

Automated breast DCE-MRI segmentation has largely been developed and evaluated in-distribution, with progress driven by expressive encoder–decoder architectures and multi-scale aggregation [4,5,6,7,42]. Curated single-institution or public datasets have enabled strong reported Dice scores, but these establish upper bounds under matched train/test distributions rather than deployment behavior. The public availability of large multi-center resources such as MAMA-MIA [3], which aggregates four TCIA collections [43,44,45,46,47], has made systematic cross-center evaluation feasible.

### 2.2. Cross-Center Generalization and External Validation

The growing literature quantifies the degradation that segmentation models incur across scanners and protocols [15,18,19]. Methodological reviews emphasize that pooled metrics can mask site-specific failure and that external validation is a prerequisite for deployment claims [16,48,49]. For breast DCE-MRI, the balanced leave-one-center-out study on the same four MAMA-MIA centers reported that a model performing well on mixed-center random splits can still lose substantial accuracy on completely unseen centers [21]. That study establishes the domain-shift problem; the present work adds low-resource adaptation and a reliability-aware routing and referral layer on top of it.

### 2.3. Few-Shot Transfer Learning

Few-shot fine-tuning adapts a source model with a small number of target labels and is the simplest adaptation a clinical team can perform [30,50]. Self-supervised and prototype-based few-shot methods reduce the target label requirement further [31]. Transfer learning has also supported data-efficient real-time visual recognition pipelines outside medical imaging, including YOLO-based systems [51]; such results illustrate the general practicality of transfer learning but are not directly comparable with volumetric DCE-MRI segmentation. In our setting, five labeled target cases represent a deliberately austere budget, and because the identity of the five cases can strongly influence the result, we redraw them in every seed.

### 2.4. Teacher–Student Learning and Pseudo-Label Quality

Self-training uses a source-trained teacher to produce pseudo-labels on unlabeled target data and trains a student to reproduce them, optionally with a small labeled set [27,28,29,52]. It is attractive clinically because it leaves the inference architecture unchanged and scales with unlabeled data. Its central risk is pseudo-label noise, which can be mitigated by confidence filtering and by anchoring the student with genuine labels [53]. We adopt confidence-filtered pseudo-labels and a supervised anchor.

### 2.5. Dynamic Model Selection

Choosing, for each input, which of several trained models to trust is the subject of dynamic classifier selection, whose methods estimate the local competence of each candidate and whose “oracle” upper bound selects the correct candidate whenever one exists [54]. AdaSwitch3D is a simple instance of this idea applied to volumetric segmentation: a logistic router estimates, from label-free prediction descriptors, which of two adapted segmenters is more likely to be better for a given case. We report the corresponding oracle selectors as non-deployable upper bounds.

### 2.6. Selective Prediction, Uncertainty, Referral, and Human-in-the-Loop AI

Selective prediction allows a model to abstain on low-confidence inputs, trading coverage for reliability [22,23]. Uncertainty estimation via Bayesian approximation or ensembles supports abstention and referral [32,33], and uncertainty-based referral has improved reliability in clinical detection tasks [24]. Calibration is a related prerequisite for trustworthy operating points [34,55]. Learning-to-defer approaches optimize the deferral decision directly [37,38], and automated segmentation quality control predicts failure explicitly [35]. AdaSwitch3D draws on this tradition but uses only a heuristic, label-free signal derived from two adapted models, with a validation-frozen referral threshold calibrated to a review-burden target rather than to a failure-capture objective.

### 2.7. Research Gap and Positioning

Existing work typically studies adaptation methods or referral mechanisms in isolation and frequently reports pooled metrics from a single randomization, which can hide both center-specific behavior and run-to-run variability. Few studies integrate low-resource adaptation, case-level model selection, and review prioritization into a single pipeline and then evaluate that pipeline once on untouched multi-center data under a leakage-controlled protocol, repeated across independent randomizations. The contribution of the present study lies in the system-level evaluation of adaptation, model selection, and referral under repeated cross-center domain shift, including explicit reporting of between-seed variability, oracle gaps, computational cost, and referral failure modes. AdaSwitch3D serves as an illustrative experimental testbed through which routing instability, workload–capture trade-offs, and referral failure modes are studied, rather than as the principal contribution or a preferred deployment method. The underlying components and segmentation backbone are established methods.

## 3. Materials and Methods

### 3.1. Study Overview

**Figure 1:**
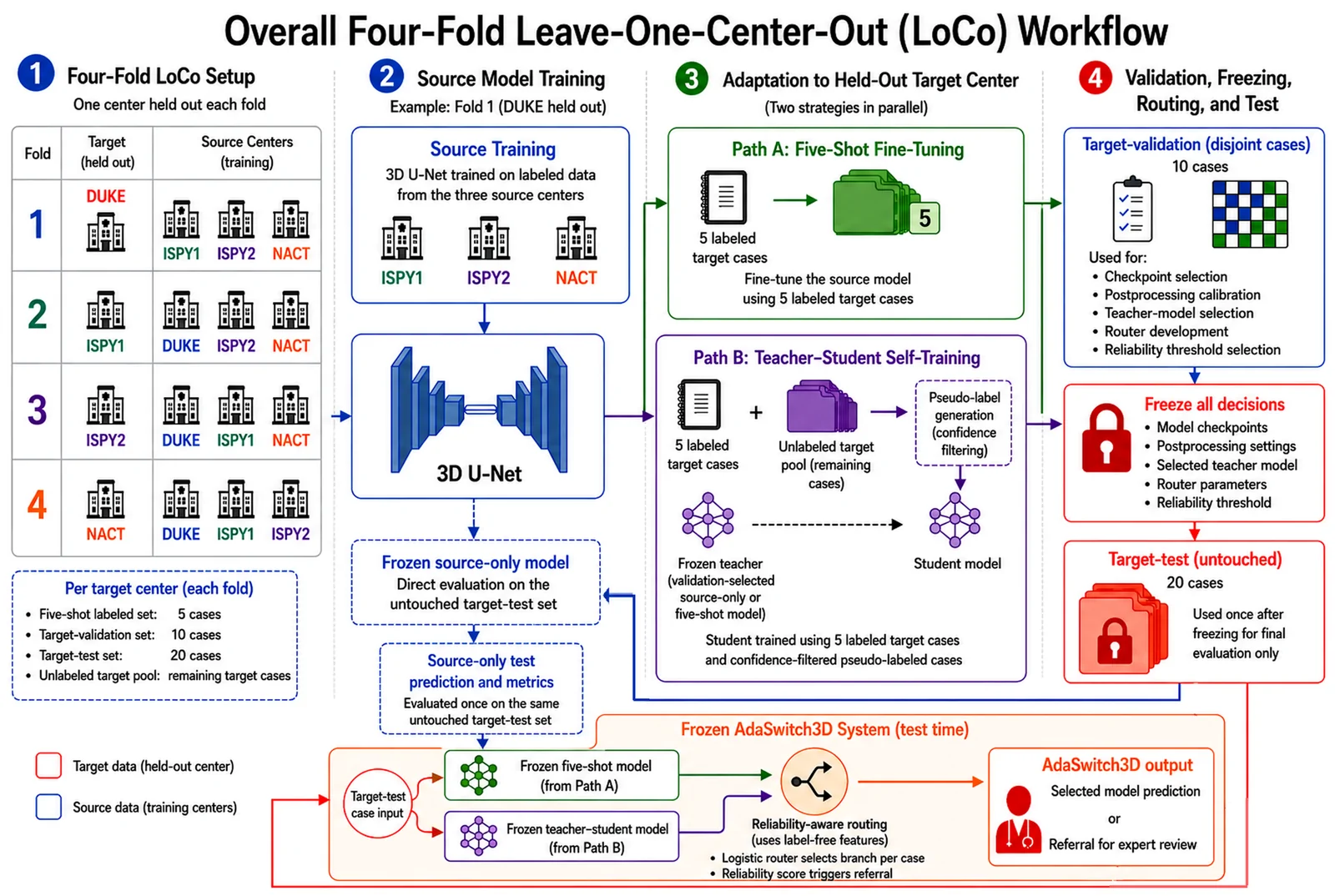
Four-fold leave-one-center-out workflow across four target centers. Arrows indicate the progression from source-only training to five-shot and teacher–student adaptation, followed by validation-only model freezing and one-time evaluation of untouched target-test cases. AdaSwitch3D routes between the two adapted branches, while heuristic review prioritization identifies cases for potential review. The entire workflow was repeated independently for five random seeds.

We evaluate three segmentation strategies and build a selection-and-referral layer on top of the two adapted models (Figure 1). AdaSwitch3D routes only between the two adapted branches, five-shot and teacher–student; the source-only model is a baseline and the student initializer, not an AdaSwitch3D inference branch. Every calibration, threshold, checkpoint, and routing decision is made using source or target validation data only, and the target test data of each seed is consumed exactly once. The complete pipeline was evaluated across five independent random seeds (42, 123, 2024, 2025, and 2026), with all model development and calibration completed separately within each seed before its untouched test evaluation (Section 3.13). The analysis structure is summarized in Table 1.

### 3.2. Dataset and Four-Center Setup

We used the four public collections aggregated in the MAMA-MIA breast DCE-MRI dataset [3]: DUKE, ISPY1, ISPY2, and NACT, all sourced from The Cancer Imaging Archive. The centers differ in scanner vendor, field strength, fat-suppression technique, number and timing of post-contrast phases, and acquisition matrix. A total of 1231 cases were used. Expert tumor segmentations provided with MAMA-MIA served as reference annotations throughout; we refer to these as expert reference annotations, not as perfect ground truth, and no independent re-annotation or adjudication was performed in this study.

### 3.3. Center-Held-Out Split and Leakage Prevention

For each of the four folds, one center is the target and the remaining three form the source pool. Within the target center, cases are partitioned into disjoint roles: five labeled cases for few-shot adaptation; ten target-validation cases for checkpoint selection, postprocessing calibration, and router development; twenty untouched target-test cases; and all remaining target cases as unlabeled data. Eight source-validation cases are drawn from each of the three source centers (24 per fold). This partition was generated independently for each of the five seeds, so the five labeled cases, the validation cases, and the test cases differ between seeds; the role sizes were identical in every seed. Fold composition for seed 42 is given in Table 2. No target-test case was used for training, pseudo-label generation, checkpoint selection, threshold tuning, postprocessing calibration, router development, or model selection; within each seed, all 80 test cases (20 per center) were evaluated once after every checkpoint, threshold, postprocessing setting, and the router were frozen.

### 3.4. Preprocessing and Input Channels

Each DCE-MRI case was converted into a standardized three-channel volume before training, adaptation, and inference. The three input channels corresponded to the pre-contrast phase and the first two post-contrast phases from the dynamic DCE-MRI series. All volumes were reoriented to a common RAS orientation and resampled to a target voxel spacing of $2.0\times 0.8\times 0.8\;{\mathrm{mm}}^{3}$. Image volumes were resampled using linear interpolation, whereas expert reference masks were resampled using nearest-neighbor interpolation to preserve discrete label values.

After spatial standardization, a foreground crop was applied to remove non-informative background. Image intensities were harmonized on a per-channel basis by clipping to the 1st–99th percentile range and scaling the clipped values to [0,1]. Expert reference annotations were binarized into foreground tumor and background classes before model training and evaluation. All preprocessing decisions were fixed before model training and were applied consistently across all data roles. The segmentation models used three input channels and were trained with patches of 80×128×128 voxels.

### 3.5. 3D U-Net Backbone

The segmentation backbone is a MONAI 3D U-Net [6,56] with three input channels and a single output channel with a sigmoid activation. Feature widths are (16,32,64,128) with strides (2,2,2), two residual units per resolution, and batch normalization. We deliberately use a standard, fixed backbone: the study concerns adaptation and system behavior, not backbone design. Hyperparameters for all three training stages are summarized in Table 3.

### 3.6. Source-Only Training

The source-only model was trained on the three source centers with the loss DiceCELoss(sigmoid=True, squared_pred=True), a learning rate of $1\times 10^{-4}$, weight decay $1\times 10^{-5}$, center-balanced sampling, a foreground sampling probability of 0.90, 250 steps per epoch, a maximum of 80 epochs, and early stopping with patience 12. Checkpoint selection used source-validation performance. This model represents naive deployment: trained elsewhere and applied directly to the target center.

### 3.7. Five-Shot Adaptation

The five-shot model was initialized from the fold-specific source-only checkpoint and fine-tuned on exactly the five labeled target cases with a learning rate of $1\times 10^{-5}$, 75 steps per epoch, a maximum of 20 epochs, and patience 5. The checkpoint and postprocessing were selected on the disjoint ten-case target-validation set. No unlabeled target data were used.

### 3.8. Teacher–Student Adaptation

For the teacher–student branch, the source-only and five-shot candidates were compared on target validation and the stronger candidate was frozen as the teacher; the teacher was therefore not always the source-only model, and the selected teacher differed by fold. The teacher generated confidence-filtered pseudo-labels on the target-unlabeled pool using a binary pseudo-label threshold of 0.50, a confident-foreground threshold of 0.90, and a confident-background threshold of 0.10; cases without confident foreground were skipped. The student was initialized from the source-only checkpoint and trained with a supervised/pseudo sampling mix of 0.50/0.50, a supervised loss weight of 1.00, a pseudo-label loss weight of 0.30, a learning rate of $1\times 10^{-5}$, 100 steps per epoch, a maximum of 20 epochs, and patience 5. The combined objective is

(1) $$L_{\mathrm{TS}}=\lambda_{\sup}\;L_{\mathrm{DiceCE}}\;\left(f_{S}\left(x^{l}\right),y^{l}\right)+\lambda_{\mathrm{pl}}\;L_{\mathrm{DiceCE}}\;\left(f_{S}\left(x^{u}\right),{\hat{y}}^{u}\right).$$

Here $f_{S}$ is the student model; $\left(x^{l},y^{l}\right)$ is a labeled target sample and its expert label from the five-shot set; $x^{u}$ is an unlabeled target sample; ${\hat{y}}^{u}$ is the confidence-filtered teacher pseudo-label for $x^{u}$; and $\lambda_{\sup}=1.00$ and $\lambda_{\mathrm{pl}}=0.30$ are the supervised and pseudo-label loss weights. The checkpoint and postprocessing were selected on target validation. Because the five-shot model is one of the teacher candidates, training the teacher–student branch already requires training the five-shot branch.

### 3.9. Postprocessing Calibration

Postprocessing was calibrated separately for each fold and method using only the ten disjoint target-validation cases, before the untouched target-test cases were evaluated. Calibration selected a probability threshold and connected-component filtering settings. The connected-component step retained the largest predicted component and removed smaller isolated components according to the frozen validation-calibrated minimum-volume setting. No morphological hole filling was applied. The frozen seed-42 per-fold, per-method probability thresholds are reported in Supplementary Table S1.

### 3.10. AdaSwitch3D Routing Features

**Figure 2:**
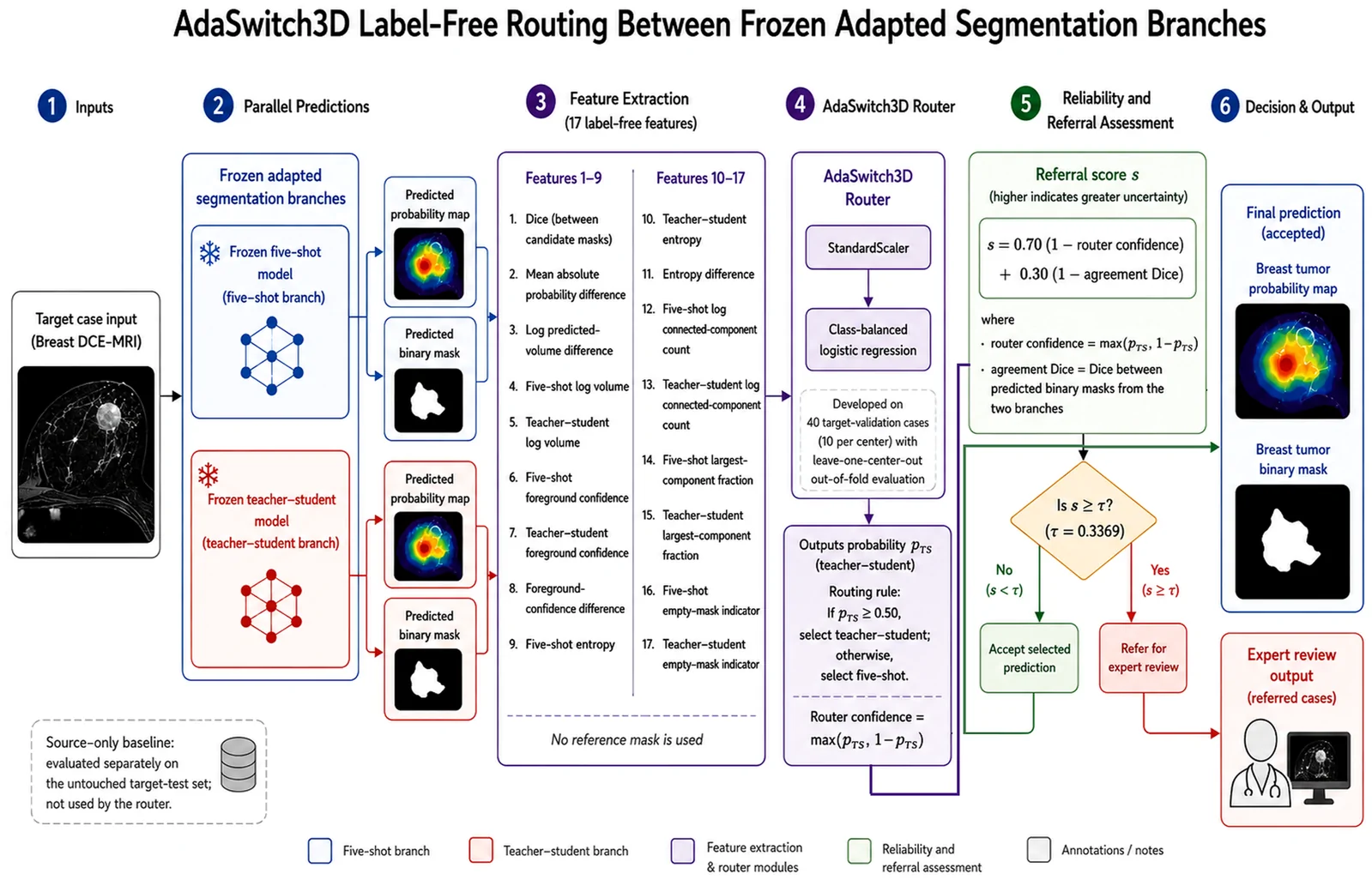
AdaSwitch3D system architecture. The five-shot and teacher–student branches are both executed; seventeen label-free features are extracted from their predictions; a validation-trained logistic router selects one branch per case; and a heuristic reliability score, combining router confidence and inter-model agreement, produces a review-prioritization flag.

AdaSwitch3D was built after the three segmentation strategies were frozen. For each case it runs the five-shot and teacher–student branches and extracts seventeen label-free features from the two predicted probability maps and masks: (1) agreement Dice between the candidate masks; (2) mean absolute probability difference; (3) log predicted-volume difference; (4) five-shot log volume; (5) teacher–student log volume; (6) five-shot foreground confidence; (7) teacher–student foreground confidence; (8) foreground-confidence difference; (9) five-shot entropy; (10) teacher–student entropy; (11) entropy difference; (12) five-shot log connected-component count; (13) teacher–student log connected-component count; (14) five-shot largest-component fraction; (15) teacher–student largest-component fraction; (16) five-shot empty-mask indicator; and (17) teacher–student empty-mask indicator. None of these features uses the reference mask. The router is a StandardScaler followed by a class-balanced logistic regression, developed on the forty target-validation cases (ten per center) with leave-one-center-out out-of-fold evaluation. The router outputs $p_{\mathrm{TS}}$, the probability that the teacher–student branch should be selected. AdaSwitch3D selects the teacher–student branch when $p_{\mathrm{TS}}\geq 0.50$ and the five-shot branch otherwise (Figure 2). The router was re-estimated from scratch in every seed. With 17 features and 40 calibration cases per seed, the router is high-dimensional relative to its training sample, which is a recognized source of instability.

### 3.11. Reliability Score and Referral

The per-case reliability (referral) score *s* combines router confidence and candidate-mask agreement:

(2) $$s=0.70\;\left(1-c_{\mathrm{router}}\right)+0.30\;\left(1-{\mathrm{Dice}}_{\mathrm{agree}}\right),$$

where $c_{\mathrm{router}}=\max\left(p_{\mathrm{TS}},1-p_{\mathrm{TS}}\right)$ is the router confidence and ${\mathrm{Dice}}_{\mathrm{agree}}$ is the agreement Dice between the five-shot and teacher–student candidate masks. A larger *s* indicates greater uncertainty. Cases with s≥τ are referred for human review. In each seed, τ was selected on that seed’s target-validation set so that 20% of validation cases would be referred, and it was not adjusted after test evaluation (for seed 42, τ=0.3369). The score is a heuristic: its weights were fixed before test evaluation and were not derived from a model of segmentation failure, and the threshold targets a review burden rather than a failure-capture objective. We therefore treat referral as a review-prioritization signal and evaluate, rather than assume, its relationship with segmentation failure.

### 3.12. One-Time Untouched Test Protocol

After all training, calibration, thresholds, and the router were frozen for a given seed, the 80 target-test cases (20 per center) of that seed were evaluated exactly once. No test case informed any modeling or calibration decision.

### 3.13. Multi-Seed Stability Analysis

To quantify sensitivity to data partitioning, few-shot case selection, and training stochasticity, the complete experimental pipeline was repeated independently with five random seeds: 42, 123, 2024, 2025, and 2026. For every seed we regenerated the target-center role assignment (including a new set of five labeled cases, new validation cases, and new test cases) and the source-validation selection; retrained the source-only model; repeated five-shot fine-tuning and teacher–student adaptation; recalibrated postprocessing on validation; rebuilt the AdaSwitch3D router; recalibrated the referral threshold; froze all components; and evaluated the untouched test cases of that seed exactly once. No hyperparameter, feature, threshold rule, or design choice was changed between seeds, and no parameter was re-estimated using test labels. All five evaluations were completed and locked before the results were aggregated.

Each seed comprised four leave-one-center-out folds with 20 test cases per center, giving 80 test cases per seed and 400 seed-specific test observations in total. Because test cases were redrawn in each seed, the same patient can appear in the test set of more than one seed; the 400 observations are therefore not 400 unique patients. For each strategy we summarize the seed-level mean Dice by the mean and sample standard deviation (SD; denominator n−1) across the five seeds and by the range. For AdaSwitch3D we additionally report the per-seed paired difference from each comparator and the number of seeds in which that difference was positive. Seed-level repetitions quantify procedural stability of the whole pipeline; with only five seeds that share the same four centers and overlapping case pools, they are not treated as independent samples for significance testing [41].

### 3.14. Referral Stability Analysis

For each seed we recorded the observed test referral rate (fraction of the 80 test cases with s≥τ); the mean Dice of automatically accepted and of referred cases (descriptive subset statistics only); the number of AdaSwitch3D test cases with Dice <0.20 (“low-Dice” cases); the number of those cases that were referred; the low-Dice capture sensitivity (referred low-Dice cases divided by all low-Dice cases); and the review precision (referred low-Dice cases divided by all referred cases). The Dice <0.20 cut-off is a descriptive definition of a poor segmentation and is not a clinically validated failure criterion. These referral summaries are used only as descriptive stability measures; they do not influence model selection, routing, or threshold calibration. As an additional post hoc descriptive analysis, we examined whether the frozen referral rule preferentially retained observations with relatively high segmentation overlap. AdaSwitch3D Dice thresholds of 0.50, 0.60, 0.70, and 0.80 were evaluated retrospectively across the 400 seed-specific test observations. For each threshold, we calculated the fraction of observations meeting the threshold, the proportion of those observations that were automatically accepted (good-case acceptance), and the proportion of all accepted observations that met the threshold (accepted-case precision). These analyses did not influence model development, calibration, routing, or referral decisions. Dice ≥0.70 was treated only as an illustrative retrospective threshold and not as a clinically validated acceptance criterion (Supplementary Table S9).

### 3.15. Sensitivity Analysis Procedures

Three additional sensitivity analyses were performed without changing any frozen test prediction. First, referral operating points were re-estimated from validation scores only. In addition to review-burden targets of 10%, 30%, 40%, and 50%, we evaluated validation thresholds chosen to capture approximately 50%, 70%, or 90% of validation cases with Dice <0.20. Each threshold was then applied unchanged to that seed’s already-locked test scores, and review burden, low-Dice capture sensitivity, review precision, and accepted-case Dice were summarized descriptively. The original frozen 20% burden threshold remains the primary operating point reported in Table 4; the sensitivity sweep is not a retrospective replacement for it. Center-specific thresholds were not estimated because each center contributed only ten validation cases per seed, making center-level operating-point estimates too discrete for a reliable comparison.

Second, router complexity was examined on validation data only by comparing the original 17-feature logistic router with predefined reduced 5-feature and 3-feature variants and stronger $L_{2}$ regularization. No test labels were used for feature selection, regularization, or router replacement, and the frozen 17-feature router remained the test-time model. Third, missed low-Dice test observations were characterized post hoc using the already-computed label-free router features to determine whether failure was associated with inter-model agreement, router confidence, predicted-volume similarity, or empty predictions. This descriptive failure analysis did not alter any threshold or prediction.

### 3.16. Metrics

For each test case, we report the Dice similarity coefficient (DSC) [57] as the primary outcome, and the Jaccard index (intersection over union, IoU), recall (sensitivity), and precision (positive predictive value) as secondary overlap outcomes. Boundary disagreement was measured using the 95th-percentile Hausdorff distance (HD95) [58], computed as the 95th percentile of the bidirectional surface distance between the predicted segmentation and the expert reference annotation. We additionally report the complete-failure rate (Dice =0), the rate of Dice <0.20, and per-case inference time. Metric choices follow current image-analysis validation recommendations [59].

HD95 units. HD95 is reported in voxel units and is treated only as a secondary, technical boundary metric for relative comparisons within this study. We audited the frozen seed-42 test records specifically for physical spacing. A spacing triplet was present for all 80 cases, but the serialized records did not preserve the axis-order/provenance needed to map that triplet unambiguously to the prediction-array axes or to certify consistency with the documented preprocessing grid. A physically valid millimeter HD95 therefore could not be reconstructed from the frozen artifacts without regenerating predictions and surface coordinates from the original spatial metadata. We consequently did not relabel or rescale voxel HD95 as millimeters. Because the preprocessing grid is anisotropic, a single multiplicative conversion would in any case be invalid. Voxel-based HD95 is not clinically equivalent to a physical distance and is not used to support a clinical claim. Finite HD95 values were summarized separately because empty predicted or reference masks yield undefined distances. Secondary metrics, including HD95, are reported for seed 42 only; the spacing audit is documented in the Supplementary Materials.

### 3.17. Statistical Analysis

Case-level analysis (seed 42; prespecified). Paired comparisons of case-wise Dice across strategies used two-sided Wilcoxon signed-rank tests [60] with Holm correction for multiple comparisons [61]. Confidence intervals for means and mean differences were obtained from 5000 center-stratified bootstrap resamples [62]. Failure-rate outcomes (complete failure and Dice <0.20) were compared with exact paired tests on discordant case pairs (McNemar/binomial), with Holm correction applied within each failure definition. Significance was assessed at α=0.05 after correction. We report raw and Holm-adjusted *p*-values and do not infer significance from non-overlapping point estimates or unadjusted *p*-values. A non-significant comparison is interpreted as not providing evidence of a difference at the prespecified level; it is not interpreted as evidence of equivalence.

Seed-level stability analysis (five seeds). Five-seed results are summarized descriptively (mean ± SD, range, and direction of paired differences) as described in Section 3.13. We did not apply significance tests to the five seed-level values, and we did not pool the 400 seed-specific observations into a single case-level test, because observations from different seeds involve different trained models and can involve the same patients. Case-level paired testing (seed 42) and seed-level stability analysis (five seeds) therefore answer different questions: the former asks whether strategies differed on one frozen evaluation; the latter asks how much the whole pipeline’s outcome varies when it is re-run.

### 3.18. Computational Cost Measurement

Inference time was measured per case as model-evaluation time on the evaluation GPU during the seed-42 test evaluation. AdaSwitch3D requires inference from both adapted branches followed by feature extraction and routing, so its inference time is expected to be approximately twice that of a single adapted model; this is a deployment trade-off rather than a property that varies with the seed. Timing was not re-measured for the additional seeds, whose architecture and inference settings were identical.

### 3.19. Implementation and Reproducibility

Models were implemented in PyTorch [63] 2.7.1+cu118 under Python 3.9.16 with CUDA 11.8 and MONAI version 1.6.1 [56], and were trained and evaluated on a single NVIDIA Tesla V100-SXM2 32 GB GPU. Random seeds 42, 123, 2024, 2025, and 2026 were used for the five complete repetitions. Inference used sliding-window aggregation with Gaussian weighting and 0.25 overlap. Frozen checkpoints, split manifests, validation-only calibration artifacts, and the one-time untouched-test protocol together define the reproducible pipeline. The exact package environment is reported in the requirements file accompanying the code repository.

## 4. Results

We first report the five-seed repeatability results (Section 4.1 and Section 4.2), which constitute the primary evidence on stability, and then the detailed, prespecified case-level analysis of seed 42 (Section 4.4).

### 4.1. Five-Seed Repeatability and Stability

**Figure 3:**
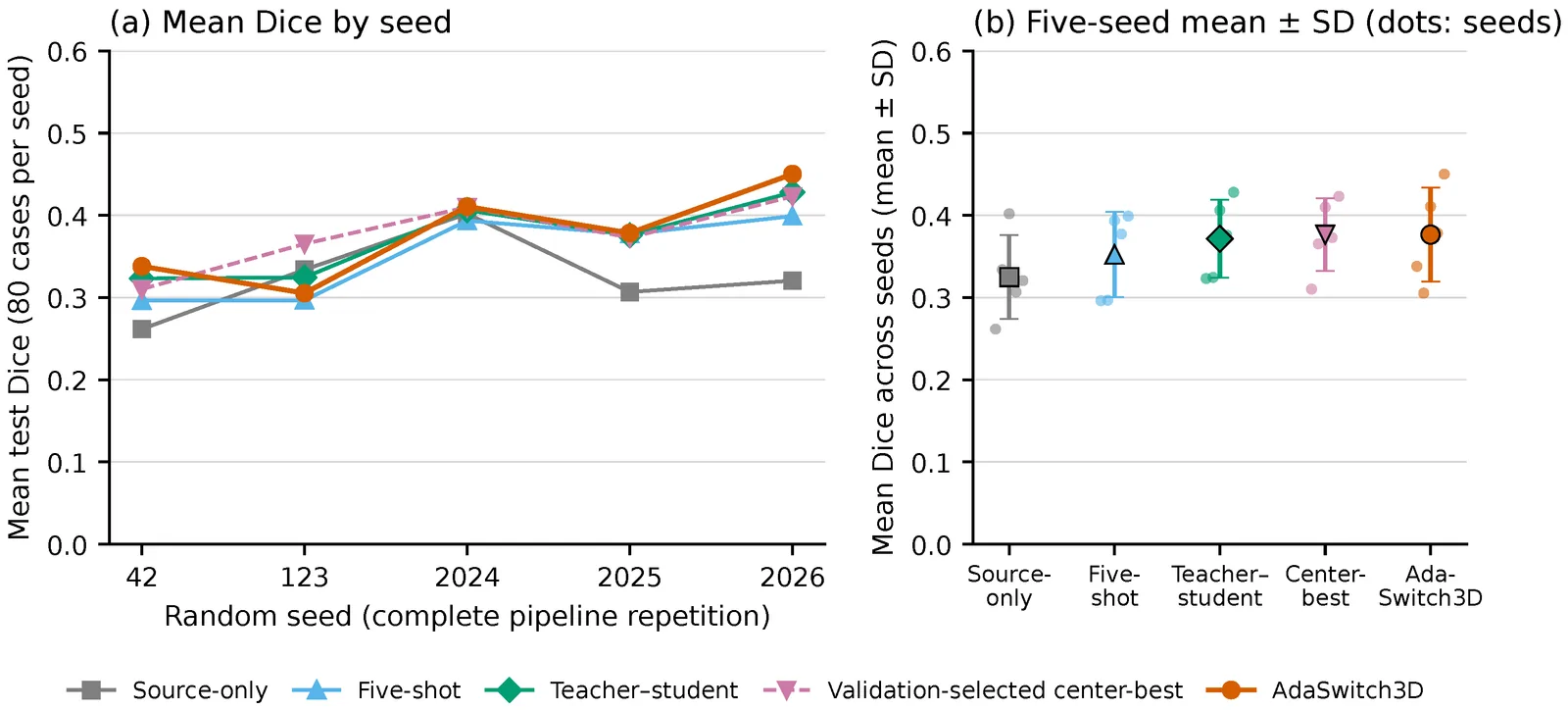
Five-seed method stability. (**a**) Mean test Dice of each deployable strategy in each seed (80 cases per seed). (**b**) Mean ± SD across the five seeds; small dots show the individual seeds. The y-axis starts at zero. Between-seed variation is larger than most between-method differences.

Table 5 and Figure 3 report mean test Dice for every strategy in each seed. Across the five independent repetitions, mean Dice was 0.3249±0.0509 for source-only transfer, 0.3525±0.0518 for five-shot fine-tuning, 0.3715±0.0474 for teacher–student adaptation, 0.3763±0.0442 for the validation-selected center-best strategy, and 0.3765±0.0573 for AdaSwitch3D. The non-deployable oracle adapted-model and all-model selectors reached 0.4130±0.0506 and 0.4615±0.0436, respectively. Absolute Dice varied substantially between seeds for every strategy (for AdaSwitch3D, from 0.3053 in seed 123 to 0.4502 in seed 2026), so the between-seed variability was larger than most between-method differences.

**Figure 4:**
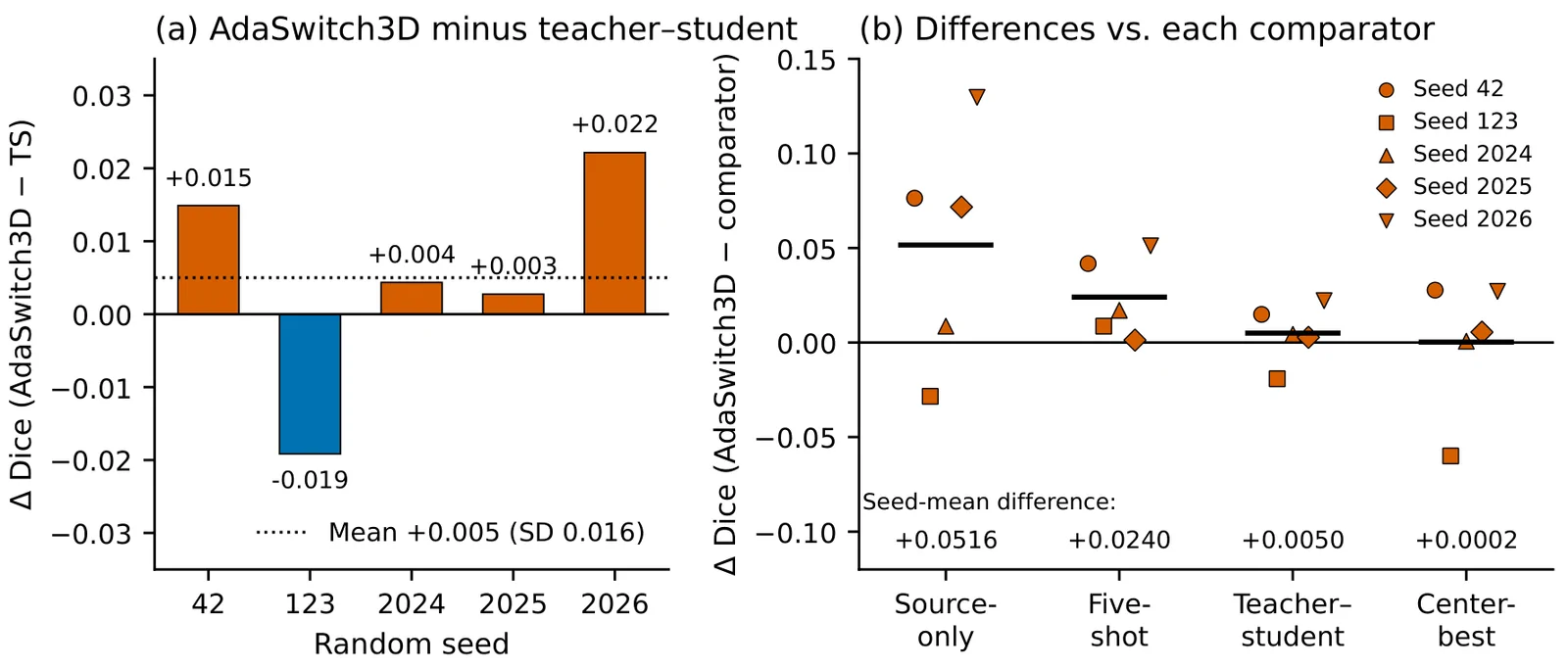
Incremental benefit of AdaSwitch3D. (**a**) AdaSwitch3D minus teacher–student mean Dice in each seed, with orange bars indicating positive differences favoring AdaSwitch3D and blue bars indicating negative differences favoring teacher–student adaptation. The horizontal zero reference line and dotted five-seed mean line are shown. Seed 123 favored teacher–student adaptation. (**b**) Per-seed differences between AdaSwitch3D and each comparator are shown using orange markers, with marker shapes identifying individual seeds. Black horizontal bars and the values below them indicate the five-seed mean differences.

Per-seed paired differences are given in Table 6 and Figure 4. AdaSwitch3D exceeded five-shot fine-tuning in all five seeds (mean difference +0.0240, range +0.0013 to +0.0511), exceeded source-only transfer in four of five seeds (mean +0.0516, range −0.0284 to +0.1296), and exceeded teacher–student adaptation in four of five seeds. Across five independent repetitions, AdaSwitch3D showed a small mean advantage over teacher–student adaptation (+0.0050, SD 0.0157), but the magnitude and direction varied across seeds (range −0.0192 to +0.0221). Relative to the validation-selected center-best strategy, AdaSwitch3D was higher in four seeds but the mean difference was essentially zero (+0.0002, SD 0.0358), and in seed 123 AdaSwitch3D was 0.0600 lower.

Seed 123 was unfavorable for adaptation and for routing. In that seed, source-only transfer (0.3338) exceeded five-shot fine-tuning (0.2967), teacher–student adaptation (0.3245), and AdaSwitch3D (0.3053); only the center-best strategy (0.3653) exceeded source-only transfer, and AdaSwitch3D was lower than teacher–student adaptation by 0.0192. This seed shows that neither the benefit of adaptation nor the benefit of routing was guaranteed under a different partition and five-shot selection. The gap between AdaSwitch3D and the oracle adapted-model selector ranged from 0.0144 (seed 2026) to 0.0585 (seed 123; mean 0.0364), indicating that the router recovered only part of the available case-level selection benefit (Supplementary Figure S5). Taken together, the five-seed evidence supports the concern that the router’s contribution beyond teacher–student adaptation is modest and seed-dependent.

### 4.2. Referral Stability Across Random Seeds

**Figure 5:**
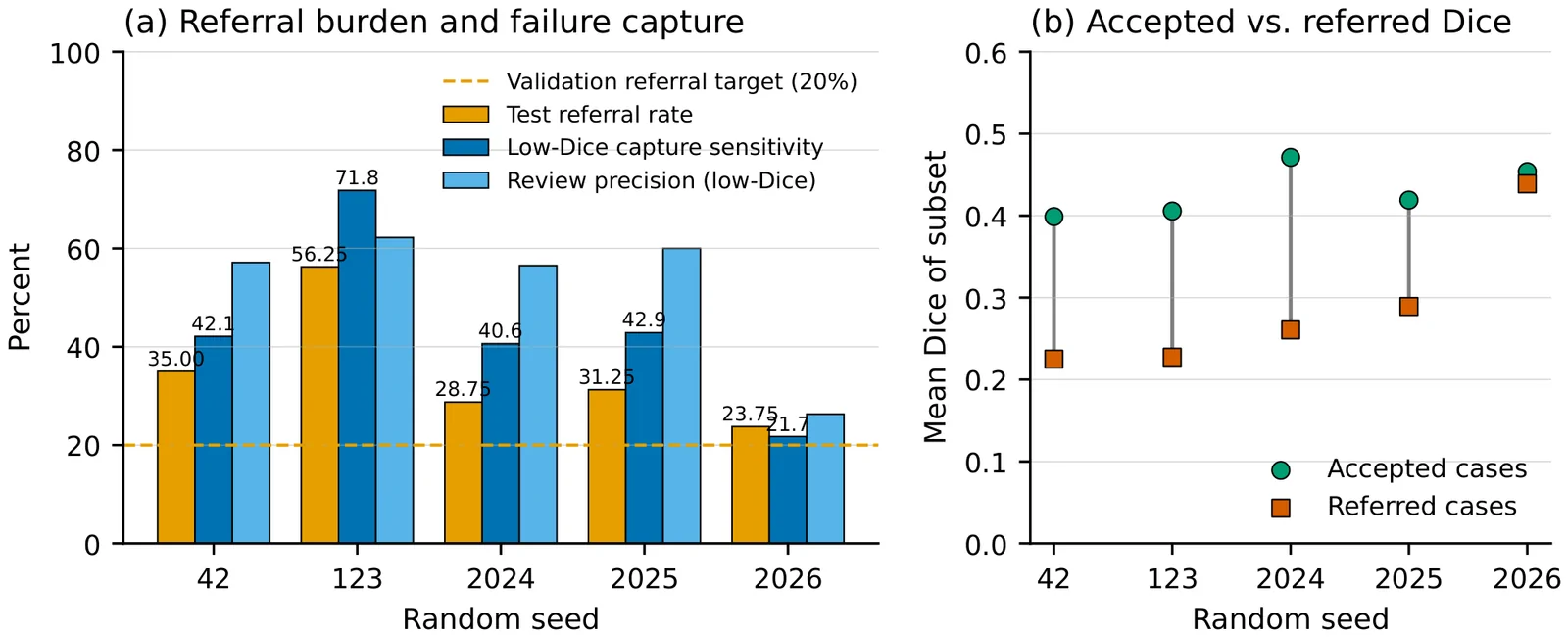
Referral stability across seeds. (**a**) Observed test referral rate, low-Dice capture sensitivity, and review precision for each seed; the dashed line marks the 20% validation referral target. (**b**) Mean Dice of automatically accepted and of referred cases in each seed. In seed 2026 the two subsets were nearly indistinguishable, and only 21.7% of low-Dice cases were referred.

Referral behavior varied substantially across seeds (Table 4, Figure 5). Although the threshold was calibrated to refer 20% of validation cases in every seed, the observed test referral rate ranged from 23.75% (seed 2026) to 56.25% (seed 123), with a mean of 35.0% (SD 12.6 percentage points); it exceeded the validation target in every seed. A review burden planned on validation data therefore did not transfer reliably to the test distribution. Low-Dice capture sensitivity ranged from 21.7% to 71.8% (mean 43.8%), and review precision from 26.3% to 62.2%. Pooled over the 400 seed-specific observations, 140 cases (35.0%) were referred, and 77 of 167 low-Dice observations (46.1%) were referred, so 90 low-Dice observations were accepted automatically. Descriptively, higher capture coincided with a higher review burden: the highest capture (71.8%, seed 123) occurred with the highest referral rate (56.25%).

Seed 2026 showed the weakest referral behavior. Only 5 of 23 low-Dice cases were referred (capture sensitivity 21.7%), fewer than one-third of referred cases had Dice <0.20 (review precision 26.3%), and the mean Dice of referred cases (0.4388) was almost identical to that of accepted cases (0.4537), so the score barely separated poor from acceptable segmentations. This occurred in the seed with the highest AdaSwitch3D Dice, indicating that segmentation accuracy and referral quality did not move together. In the other four seeds, accepted cases had a mean Dice 0.130–0.211 higher than referred cases. The referral signal therefore enriched the review queue for poor cases in most seeds but was not a consistent failure detector.

A complementary retrospective analysis evaluated whether the referral mechanism preferentially retained relatively high-quality segmentations (Supplementary Table S9). Across the 400 seed-specific observations, 97 (24.25%) achieved Dice ≥0.70. The frozen referral rule automatically accepted 77 of these 97 observations (79.4%). However, only 77 of the 260 automatically accepted observations (29.6%) achieved Dice ≥0.70. Accepted-case precision also varied across centers, ranging from 19.4% at DUKE to 39.4% at NACT. These results indicate that, although the referral rule retained a substantial proportion of high-Dice observations, it did not reliably isolate them from lower-quality segmentations, and most automatically accepted observations remained below the illustrative Dice 0.70 threshold.

### 4.3. Sensitivity Analysis Results

The referral sensitivity analysis showed a clear workload–capture trade-off rather than a stable operating point (Supplementary Table S5 and Figure S6). When validation thresholds targeted review burdens of 10%, 30%, 40%, and 50%, the mean test review burden across seeds was 23.8%, 39.8%, 48.0%, and 56.8%, respectively, while mean low-Dice capture sensitivity increased from 31.7% to 47.8%, 58.0%, and 62.9%. Validation thresholds aimed directly at 50%, 70%, and 90% low-Dice capture yielded mean test burdens of 52.5%, 71.8%, and 96.0%, with mean test capture sensitivities of 58.7%, 71.8%, and 96.9%, respectively. The corresponding accepted-case Dice means were 0.409, 0.317, and 0.198. Thus, substantially higher failure capture required reviewing a large fraction of cases, and the existing score did not provide a sharply favorable risk–coverage trade-off.

No evaluated operating point approached a high-sensitivity, low-workload regime. As an illustrative benchmark, achieving at least 90% failure capture while reviewing no more than 30% of cases would represent a substantially more favorable operating point than observed here. The lowest-workload condition produced a mean test review burden of 23.8% but captured only 31.7% of low-Dice failures, while the validation policy targeting 90% failure capture achieved 96.9% test capture only by referring 96.0% of test cases. Thus, the current referral score does not support high failure sensitivity at a practically selective review burden.

All alternative thresholds were selected from validation data only; none was tuned on test labels or substituted for the frozen primary threshold.

A validation-only router simplification check compared the original 17-feature logistic router with predefined 5-feature and 3-feature variants and stronger regularization (Supplementary Table S6). Across the tested configurations, the validation out-of-fold Dice summary was indistinguishable (mean 0.4284, SD 0.0426 across seeds). This small-sample sensitivity check provided no evidence that the full 17-feature representation improved validation performance over lower-dimensional alternatives; it also did not justify replacing the already-frozen test router.

The missed-failure analysis further explained why disagreement-based referral can fail (Supplementary Table S7 and Figure S7). Among the 90 low-Dice observations that were not referred, median inter-model agreement was 0.840 and median router confidence 0.846, compared with 0.162 and 0.655, respectively, among the 77 referred low-Dice observations. Missed failures also had a smaller median log-volume difference (0.217 vs. 0.907), and both candidate models were empty in 20 of the 90 missed low-Dice observations. These patterns show that both branches can agree and be confident while being wrong, which directly limits a referral score built from disagreement and router uncertainty.

### 4.4. Detailed Case-Level Analysis of Seed 42 (Prespecified)

The following subsections report the original, prespecified frozen evaluation of seed 42 in detail. Seed 42 is one of the five repetitions and is not representative of all seeds; for example, its AdaSwitch3D Dice (0.3380) was below the five-seed mean (0.3765).

#### 4.4.1. Validation Behavior

On the forty target-validation cases, the pooled mean Dice was 0.425 for five-shot and 0.439 for teacher–student adaptation, so neither adapted branch dominated across all centers. The branch that was strongest on validation differed by center: teacher–student was strongest for DUKE and ISPY1, whereas five-shot was strongest for ISPY2 and NACT. This heterogeneity motivated case-level selection. Under leave-one-center-out out-of-fold evaluation on validation, AdaSwitch3D reached a mean Dice of 0.461, compared with 0.425 (five-shot) and 0.439 (teacher–student), and approached the oracle adapted-model selector value of 0.481. Routing accuracy was 67.5%. Accepted cases had a mean Dice of 0.490 and referred cases 0.347. These validation estimates are based on 40 cases and were optimistic relative to the untouched test results.

#### 4.4.2. Overall Test Results

**Figure 6:**
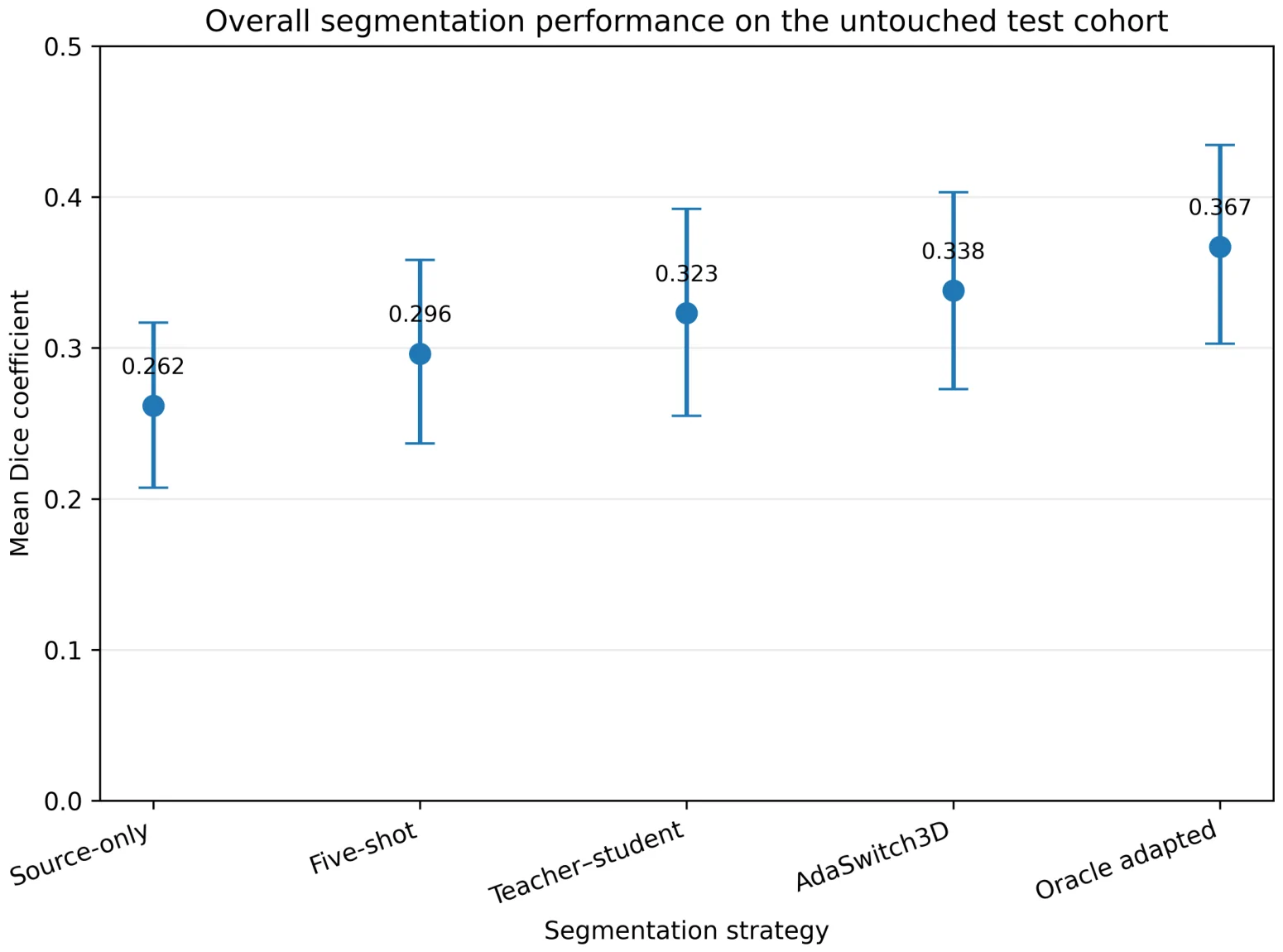
Overall mean test Dice for seed 42 with 95% center-stratified bootstrap confidence intervals for each strategy. Oracle adapted-model and all-model selectors are shown only as non-deployable upper bounds.

Table 7 reports the one-time results on the 80 untouched test cases of seed 42. Source-only transfer achieved a mean Dice of 0.262 (95% CI 0.207–0.317). Five-shot fine-tuning reached 0.296 (0.237–0.358), teacher–student adaptation 0.323 (0.255–0.392), and AdaSwitch3D 0.338 (0.273–0.403). The per-center validation-selected strategy reached 0.310. Two oracle selectors are reported as reference bounds only: the oracle adapted-model selector chooses the better of the five-shot and teacher–student predictions using the expert reference annotation and reached 0.367 (0.303–0.434), and the oracle all-model selector, which also includes the source-only model, reached 0.403. Because both require the reference annotation at inference time, neither is deployable. These patterns are summarized in Figure 6.

#### 4.4.3. Center-Wise Results

**Figure 7:**
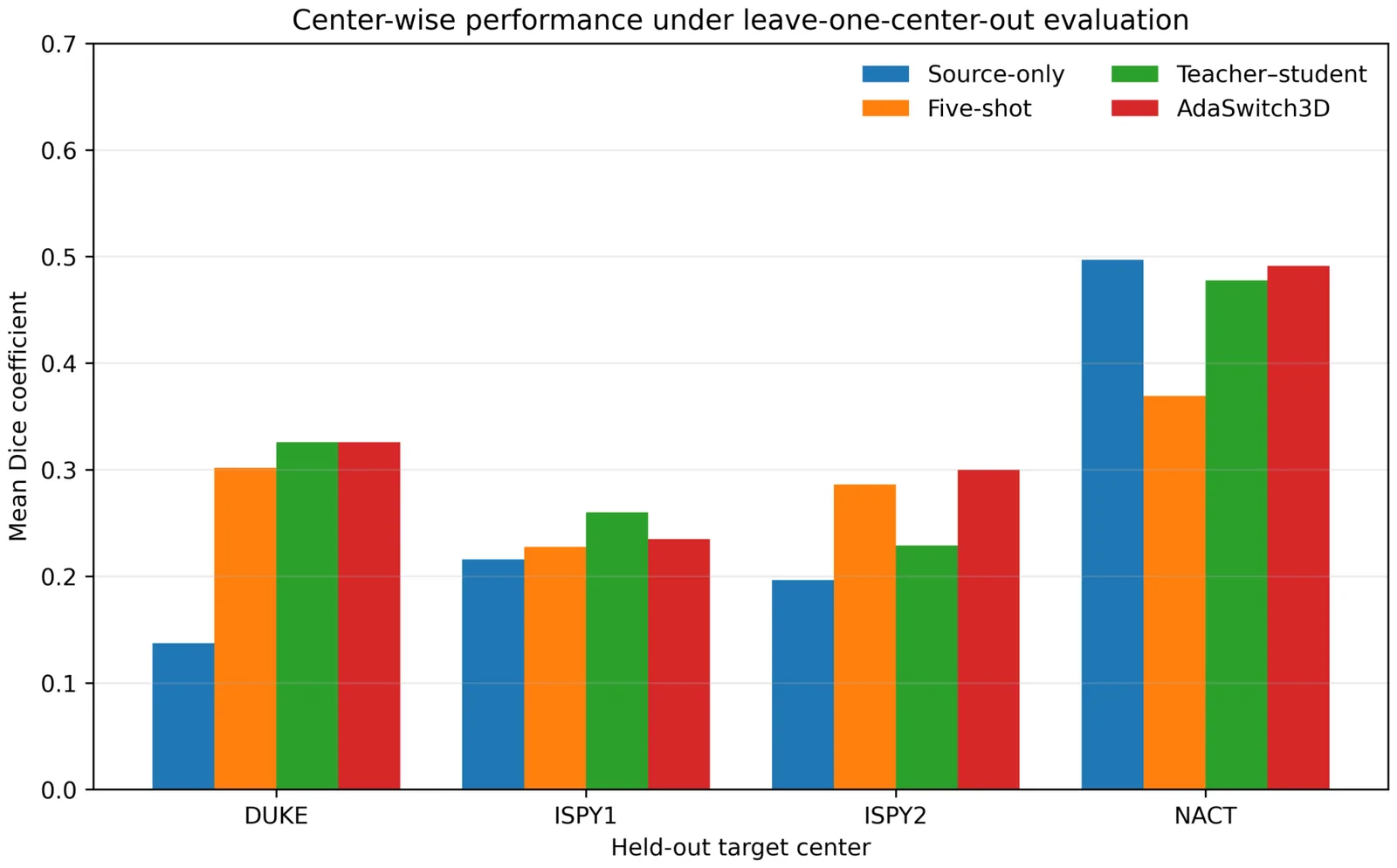
Center-wise mean test Dice for seed 42 for each strategy across DUKE, ISPY1, ISPY2, and NACT. The spread across centers illustrates residual heterogeneity and the absence of uniform improvement from adaptation.

Center-wise behavior was heterogeneous, and no single strategy improved every center (Table 8, Figure 7). On DUKE, mean Dice rose from 0.137 (source-only) to 0.302 (five-shot) and 0.326 (teacher–student and AdaSwitch3D). On ISPY1, teacher–student (0.260) exceeded AdaSwitch3D (0.235), so routing did not always select the locally best branch. On ISPY2, AdaSwitch3D (0.300) exceeded both five-shot (0.286) and teacher–student (0.229). On NACT, source-only transfer was already strong (0.497) and was not surpassed by adaptation; five-shot reduced performance to 0.369, while teacher–student (0.478) and AdaSwitch3D (0.491) remained close to the source-only value. Per-center results were analyzed in detail for seed 42 only.

#### 4.4.4. Statistical Comparisons

**Figure 8:**
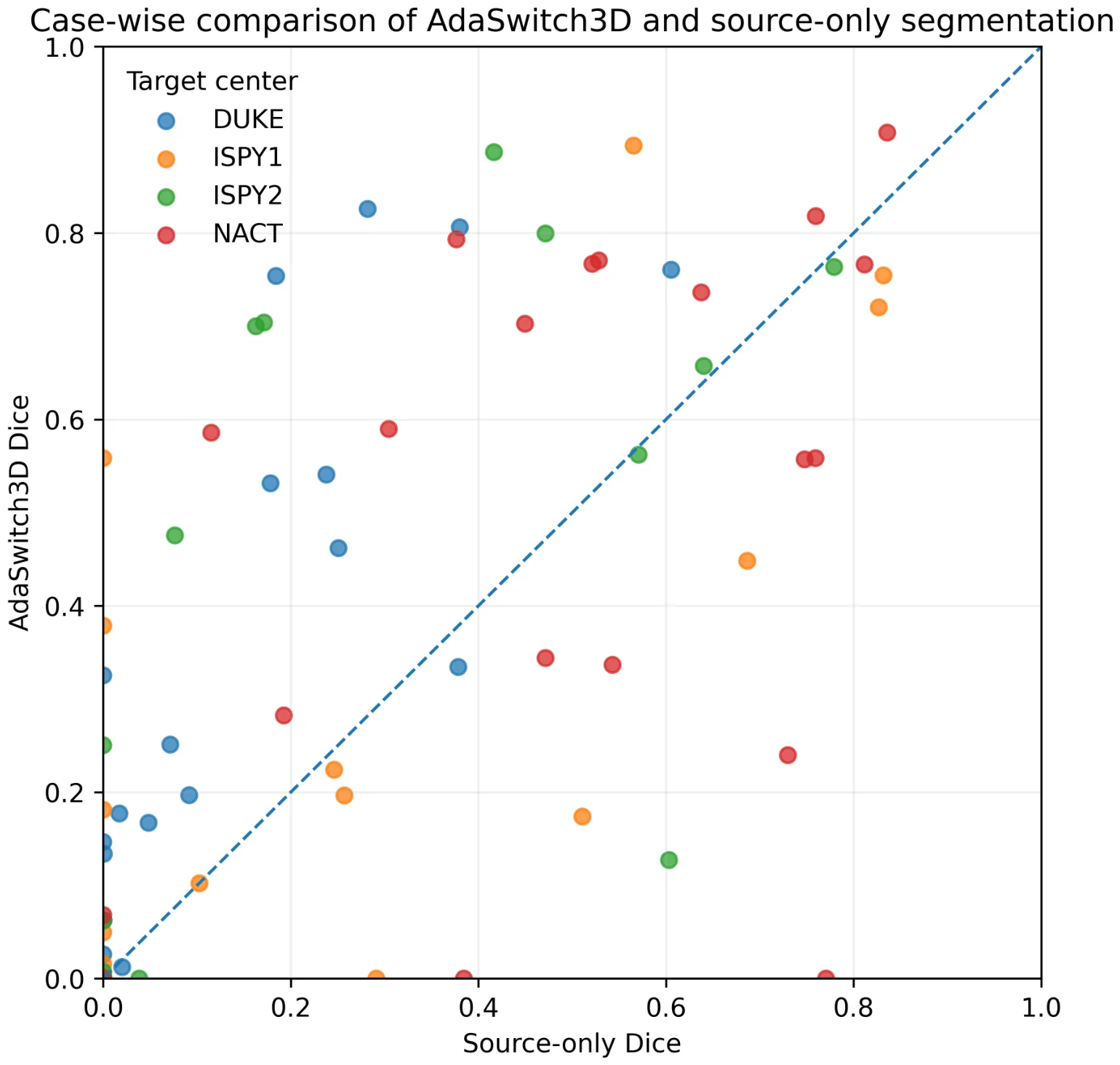
Paired case-level comparison of AdaSwitch3D against source-only transfer on the untouched test set of seed 42. Each point is a test case; the shift relative to the identity line reflects the pooled improvement (Holm-adjusted p=0.021).

Paired Wilcoxon signed-rank tests with Holm correction (Table 9) showed that AdaSwitch3D had higher Dice than source-only transfer (difference +0.076; 95% CI 0.024–0.127; raw p=0.003; Holm-adjusted p=0.021) as illustrated in Figure 8 and five-shot fine-tuning (difference +0.042; 95% CI 0.012–0.071; raw p=0.003; Holm-adjusted p=0.021). For AdaSwitch3D versus teacher–student adaptation (difference +0.015; 95% CI −0.010 to 0.043; raw p=0.255; Holm-adjusted p=0.508), the comparison did not provide evidence of a difference at the prespecified significance level; this result does not establish that the two methods are equivalent. Five-shot versus source-only (+0.035; Holm p=0.508), teacher–student versus source-only (+0.061; Holm p=0.060), teacher–student versus five-shot (+0.027; Holm p=0.274), and AdaSwitch3D versus the validation-best strategy (+0.028; Holm p=0.274) were not significant after correction. These case-level tests apply to seed 42 only; the five-seed results in Section 4.1 show that the seed-42 advantage over teacher–student adaptation was not reproduced in every seed.

#### 4.4.5. Failure Rates and HD95

In seed 42, AdaSwitch3D had a complete-failure rate of 23.75%, compared with 32.50% for source-only transfer, and a Dice-below-0.20 rate of 47.50%, compared with 55.00%. Using exact paired tests on discordant pairs with Holm correction within each failure definition, no complete-failure or Dice-below-0.20 comparison was statistically significant (Supplementary Table S2). Across the five seeds, the AdaSwitch3D Dice-below-0.20 rate ranged from 28.75% (seed 2026) to 48.75% (seed 123), with 167 of 400 seed-specific observations (41.75%) below 0.20 (Table 4).

**Figure 9:**
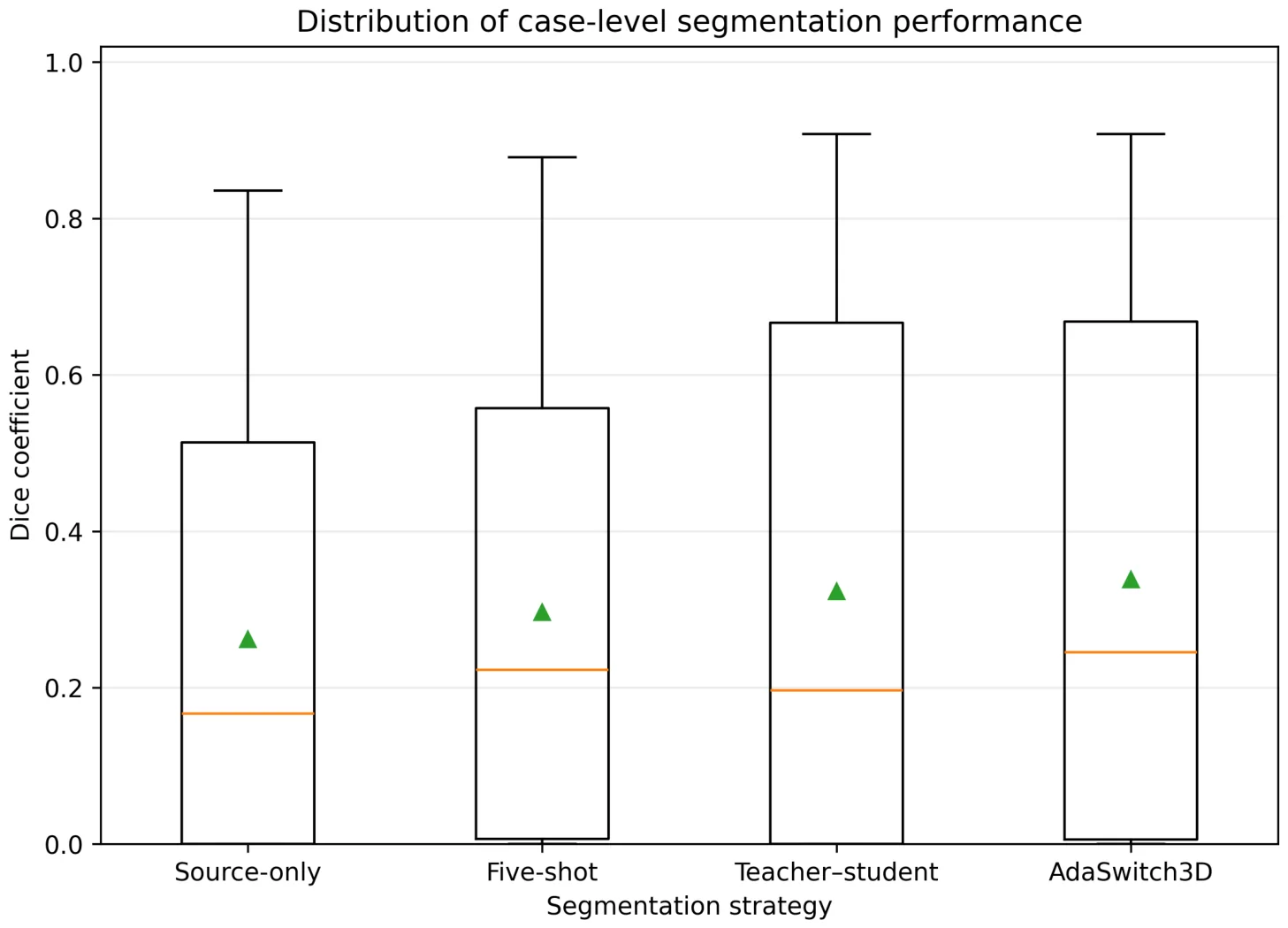
Case-level test Dice distributions by strategy for seed 42. Orange horizontal lines indicate the median Dice values, while green triangles represent the mean Dice values. Boxes show the interquartile ranges, and whiskers indicate the spread of the distributions. Adaptation and routing compress the lower tail relative to source-only transfer, but a substantial fraction of low-Dice and complete-failure cases persists under every strategy.

Median finite HD95 was lowest for teacher–student adaptation (23.246 voxels) and similar for AdaSwitch3D (26.840 voxels), both below source-only transfer (52.145 voxels). These are voxel-unit summaries on an anisotropic grid; they support only relative comparison within this study and should not be interpreted as physical boundary errors in millimeters. The case-level Dice distributions are shown in Figure 9.

#### 4.4.6. Routing and Referral in Seed 42

**Figure 10:**
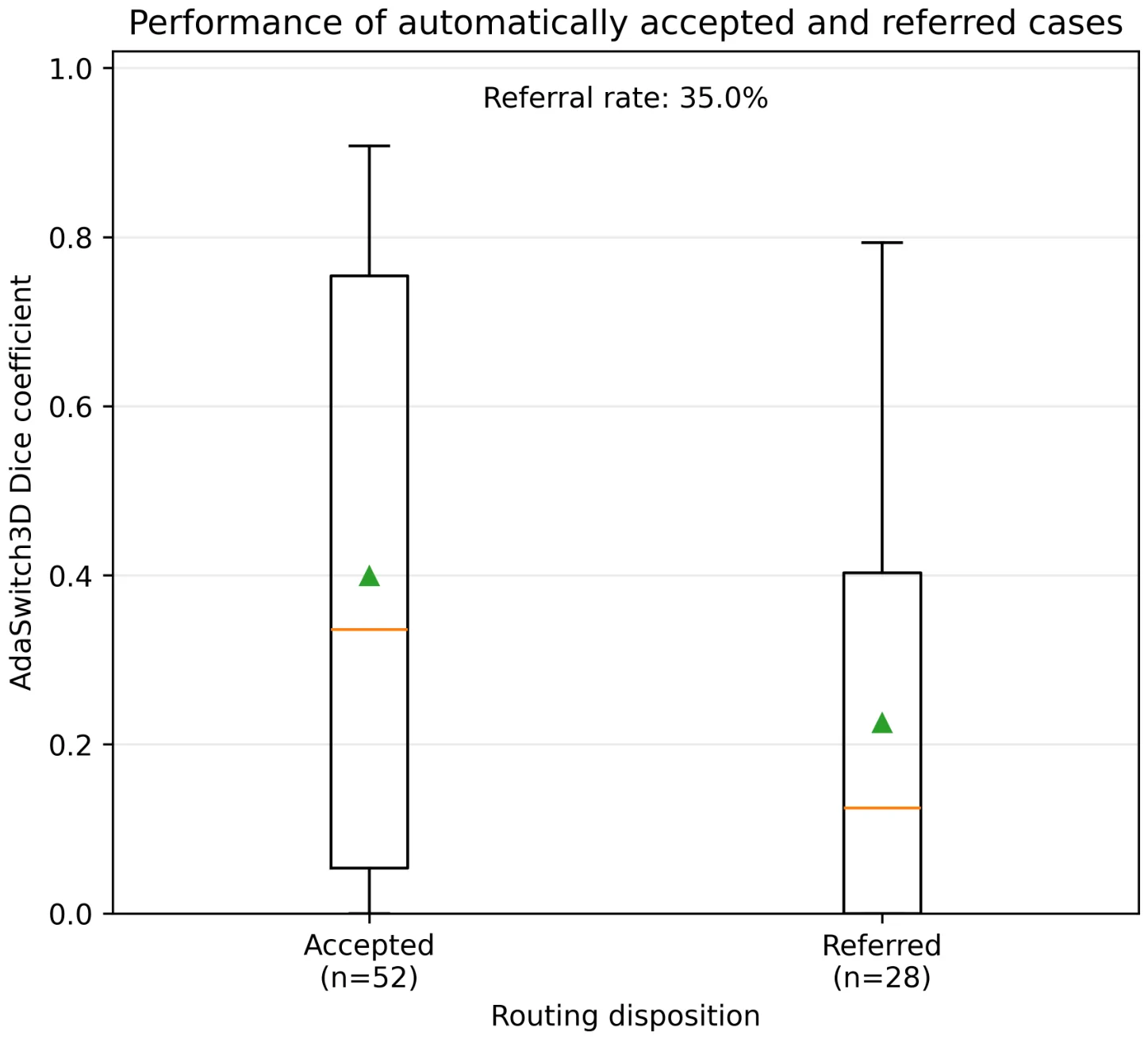
Accepted versus referred test cases under AdaSwitch3D for seed 42. Orange horizontal lines indicate median Dice values, and green triangles represent mean Dice values. Boxes show the interquartile ranges, and whiskers indicate the spread of case-level Dice scores. Automatically accepted cases (*n* = 52) had a higher mean Dice (0.399) than referred cases (*n* = 28; 0.225). The referral rate was 35.0%, above the 20% validation target; low-Dice capture sensitivity was 42.1% and review precision 57.1%. Across seeds, this separation was not consistently reproduced (Figure 5).

On the untouched test set of seed 42, AdaSwitch3D selected the teacher–student branch for 53 cases and the five-shot branch for 27 cases (Table 10). The mean router confidence was 0.751 and the mean candidate-model agreement Dice was 0.560. The referral mechanism referred 35.0% of cases (28/80), higher than the 20% validation target. The 52 automatically accepted cases had a mean Dice of 0.399, whereas the 28 referred cases had a mean Dice of 0.225 (Figure 10). Of the 38 test cases with Dice <0.20, 16 were referred (capture sensitivity 42.1%; review precision 57.1%). A post hoc threshold-transfer summary for seed 42 is given in Supplementary Table S3; the threshold was not retuned.

#### 4.4.7. Qualitative Results

**Figure 11:**
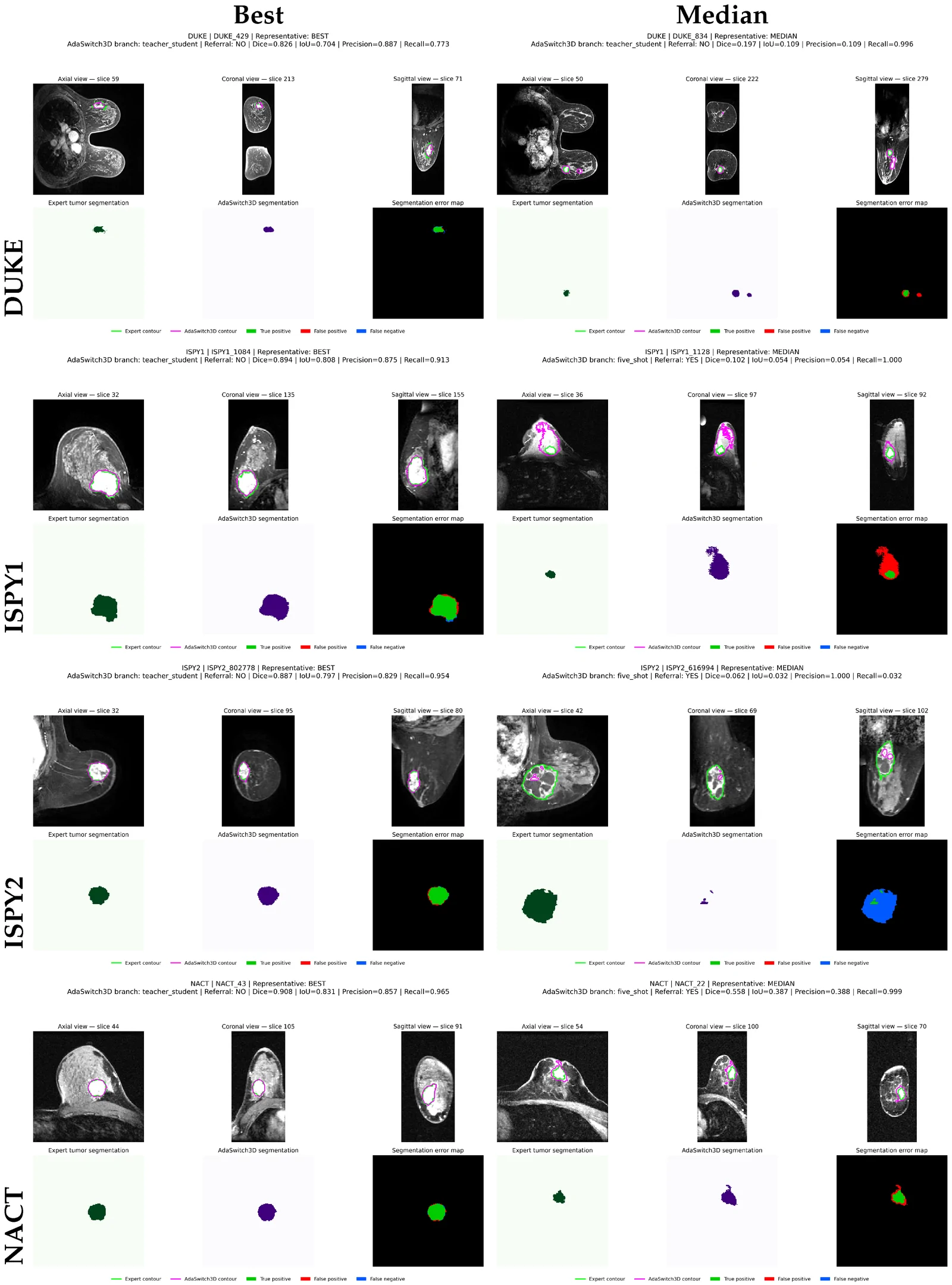
Representative 2D qualitative results by center for seed 42, showing the best and median cases selected by frozen AdaSwitch3D Dice. Panels compare expert reference and AdaSwitch3D contours with true-positive, false-positive, and false-negative regions. Worst cases and 3D renderings are provided in the Supplementary Materials.

The best and median cases by frozen AdaSwitch3D Dice for each center in seed 42 are shown in Figure 11; they were selected deterministically and are not random examples. Agreement with the expert reference annotation was high in the best cases (Dice 0.826–0.908) and degraded through under-segmentation, over-segmentation into enhancing parenchyma, and missed lesions in the median cases. The corresponding worst cases and 3D renderings are provided in the Supplementary Materials.

### 4.5. Computational Cost Results

Measured model inference in seed 42 averaged 0.090 s per case for source-only, 0.081 s for each single adapted branch and therefore also for the center-best strategy, which applies one branch per case, and 0.164 s for the full AdaSwitch3D system on the evaluation GPU, approximately twice the single-branch time because both adapted models were executed; feature extraction and routing added little beyond the two forward passes. These are model-evaluation times and do not include image loading, preprocessing, data transfer, visualization, or clinical-workflow overhead. AdaSwitch3D also requires both adapted models to be stored, maintained, and monitored, and the router and referral threshold to be recalibrated at each deployment site.

## 5. Discussion

### 5.1. What Adaptation Accomplished

A standard 3D U-Net trained on three centers performed poorly at an unseen center, and inexpensive adaptation with five labeled cases recovered part, but only part, of that loss. Across five complete repetitions, teacher–student adaptation raised mean Dice from 0.3249±0.0509 (source-only) to 0.3715±0.0474, and five-shot fine-tuning to 0.3525 ± 0.0518. The benefit was not guaranteed: in seed 123, source-only transfer exceeded both adapted models, and in seed 42 source-only transfer was already the best strategy at NACT. Adaptation under a five-case budget should therefore be regarded as a partial and seed-dependent correction of cross-center shift, not as a solution to it.

### 5.2. What the Router Added

The motivation for case-wise routing is that neither adapted branch dominated: the better branch differed by center and by case, and the oracle adapted-model selector exceeded both branches in every seed. Routing therefore targets per-case selection rather than simply maximizing mean Dice with a single model, in the tradition of dynamic classifier selection [54]. The empirical question is how much of the oracle headroom a label-free router can recover. Our answer is: little and inconsistently. Across five seeds, AdaSwitch3D exceeded teacher–student adaptation by only +0.0050 on average (four of five seeds; range −0.0192 to +0.0221), its five-seed mean was essentially identical to that of the simpler validation-selected center-best strategy (0.3765 versus 0.3763), and the gap to the oracle adapted-model selector remained 0.0144–0.0585. In the prespecified seed-42 analysis, the comparison with teacher–student adaptation did not provide evidence of a difference (Holm-adjusted p=0.508). A validation-only simplification check did not identify a measurable advantage for the full 17-feature router over reduced 3- or 5-feature variants, reinforcing that the present calibration sample is too small to justify added router complexity. We therefore do not claim that AdaSwitch3D is superior to teacher–student adaptation; the data are compatible with a small average benefit whose direction depends on the particular partition and five-shot selection. The router’s contribution is best regarded as exploratory evidence that case-wise selection is feasible, together with a quantified indication of how much remains to be gained by better selection.

### 5.3. What Multi-Seed Repetition Revealed

The five-seed repetition strengthened the assessment of repeatability and revealed limitations that were not apparent from seed 42 alone. First, between-seed variability was large for every strategy (SD 0.044–0.057 Dice), larger than most between-method differences, which is consistent with the known instability of estimates from small, heterogeneous samples [40,41]. Second, the seed-42 result, in which AdaSwitch3D was numerically best among deployable strategies, was not reproduced in every seed: seed 123 favored teacher–student adaptation, center-best selection, and even source-only transfer over AdaSwitch3D. Third, referral burden and failure capture varied far more than the fixed validation target would suggest (Section 4.2). The consistent findings were limited to the direction of a few comparisons (AdaSwitch3D above five-shot fine-tuning in all seeds; adaptation above source-only transfer on average) and the persistence of low absolute accuracy. We consider this a scientifically useful outcome: single-seed evaluation of few-shot cross-center pipelines can materially overstate both the stability and the size of incremental gains.

### 5.4. Computational Trade-Off

AdaSwitch3D requires both adapted branches at inference, roughly doubling model-evaluation time relative to a single adapted model (0.164 versus 0.081 s per case in seed 42), and it adds the operational burden of maintaining two models, a router, and a site-specific referral threshold. Training cost is less affected, because the five-shot model is already trained as a teacher candidate for the teacher–student branch. Given a small and seed-dependent accuracy difference, teacher–student adaptation—or a validation-selected single strategy per center—may be preferable where computational simplicity, latency, or maintainability is the primary deployment constraint. The absolute times are small on modern hardware, so the more important cost is complexity rather than seconds per case. More broadly, deployment-oriented real-time vision systems that combine recognition with auxiliary tracking modules illustrate why end-to-end system cost, rather than backbone inference alone, is the relevant engineering quantity [64]. Any justification for dual-branch inference would therefore have to come from a clearly larger or more consistent accuracy or reliability benefit than observed here.

Accordingly, the present results do not establish AdaSwitch3D as a preferred deployment architecture; rather, the routing experiment illustrates the difficulty of obtaining a sufficiently large and stable case-wise selection benefit to justify added system complexity.

### 5.5. Referral Instability

The referral mechanism should not be regarded as a validated safety mechanism or as a reliable detector of segmentation failure. With the threshold calibrated to a 20% validation burden in every seed, test referral ranged from 23.75% to 56.25%, and low-Dice capture ranged from 21.7% to 71.8%; overall, 90 of 167 low-Dice observations were accepted automatically. In seed 2026 the score barely separated poor from acceptable segmentations. The validation-only threshold sweep confirmed a broad workload–capture trade-off: progressively higher failure capture required reviewing a large fraction of test cases, with the 70% validation-capture policy producing a mean test burden of 71.8%. The missed-failure analysis also showed a structural limitation of the score. Missed low-Dice cases had much higher median agreement between the two candidate models (0.840) and higher router confidence (0.846) than referred low-Dice cases, while their predicted volumes were more similar; in 20 of 90 missed low-Dice observations both candidate masks were empty. Thus, two branches can be confidently and consistently wrong, leaving little disagreement signal for the referral rule to exploit. Additional contributors include the small validation sample and a heuristic score whose weights were not derived from a model of segmentation failure, in a setting where segmentation networks are known to be poorly calibrated [34]. In its current form the signal is at most a workload-oriented review-prioritization heuristic whose behavior must be verified locally; it cannot be used to decide that an automatically accepted segmentation is safe.

### 5.6. Clinical Interpretation

Absolute accuracy was low under every deployable strategy (five-seed mean Dice 0.325–0.377), and 28.75–48.75% of AdaSwitch3D segmentations per seed had Dice <0.20. Dice values of approximately 0.70 have been used as heuristic indicators of good overlap [65]; however, this value was not developed as a clinically validated threshold for breast tumor segmentation and should not be interpreted as a regulatory or task-specific acceptance criterion. Acceptable performance depends on the intended use, such as volumetry, response assessment, treatment planning, or review prioritization, and must ultimately be defined with clinical input.

To further examine the possibility of identifying relatively high-quality segmentations, we performed a retrospective analysis using Dice ≥0.70 as an illustrative threshold. Across the 400 seed-specific observations, 97 (24.25%) achieved Dice ≥0.70, and 77 of these 97 observations (79.4%) were automatically accepted by the frozen referral rule. However, only 77 of the 260 automatically accepted observations (29.6%) achieved Dice ≥0.70, with accepted-case precision ranging from 19.4% at DUKE to 39.4% at NACT (Supplementary Table S10). Thus, although the referral mechanism retained many high-Dice observations, it did not reliably isolate them from lower-quality segmentations.

Importantly, true Dice cannot be calculated prospectively because the expert reference segmentation is unavailable during deployment. A practical high-quality-case screening workflow would therefore require a separately validated segmentation-quality estimator capable of predicting contour accuracy without access to the reference annotation. The present referral signal does not provide such an estimator.

For review prioritization, the more relevant operational criterion is failure capture at an acceptable review workload. The workload–capture sensitivity analysis showed that the current heuristic does not approach a high-sensitivity, low-workload operating regime. The lowest-workload evaluated condition produced a mean test review burden of 23.8% but captured only 31.7% of low-Dice failures. Conversely, a validation policy targeting 90% failure capture achieved 96.9% capture on the test data only by referring 96.0% of test observations. As an illustrative benchmark, a system capable of capturing at least 90% of failures while referring no more than 30% of cases would represent a substantially more favorable operating point than any observed here; this benchmark is not presented as a clinically validated requirement. The present results therefore show that high failure sensitivity can be achieved only at the cost of reviewing nearly all cases, which removes the intended efficiency advantage of selective referral.

The relative improvements reported here should therefore not be equated with clinical utility. The present system is not suitable for autonomous clinical use or reliable clinical triage. Any future clinical role would require substantially better absolute segmentation accuracy, direct and validated quality-estimation or failure-detection mechanisms, independent external and prospective validation, and reader studies assessing effects on expert workload and decision making [48,49].

### 5.7. Comparison with the Literature

The center heterogeneity we observe is consistent with the balanced leave-one-center-out analysis on the same cohort [21] and with the broader finding that pooled metrics can obscure site-specific failure [16,17]. Our self-training behavior aligns with reports that a small labeled set plus a confidence-filtered pseudo-labeled pool can help when pseudo-label noise is controlled [29,53]. The referral results are consistent with evidence that deferral works best when the deferral rule is learned against the error it is meant to catch [37,38], and that per-case segmentation quality control benefits from dedicated failure predictors [35]; AdaSwitch3D used neither.

### 5.8. Future Work

The referral findings should be interpreted primarily as a negative methodological result. The present disagreement- and confidence-based heuristic did not provide a sufficiently reliable relationship between review burden and failure capture for this task. In particular, severe failures were frequently missed when the two candidate models agreed and the router was confident, indicating that further tuning of the same heuristic is unlikely to resolve the underlying failure mode.

Future referral and automatic-acceptance systems should therefore investigate fundamentally different mechanisms that estimate segmentation failure directly rather than relying primarily on inter-model agreement and router confidence. Priority directions include trained segmentation-quality or failure-detection models, learning-to-defer approaches that explicitly optimize when a case should be referred, and conformal or other risk-controlling selective-prediction methods with explicit error control [39] adapted to non-exchangeable cross-center data. Based on the observed high-agreement missed failures, trained failure detectors or segmentation-quality estimators that learn directly from segmentation error should be prioritized, while inter-model disagreement should be treated as a supplementary feature rather than as the primary referral signal.

Failure-capture or risk–coverage objectives [23] should be used to evaluate these approaches, because the present validation-only sensitivity analysis showed that high failure capture with the current score required reviewing a large fraction of cases. Referral workload should ultimately be calibrated prospectively against clinically defined error sensitivity using larger calibration cohorts and independent external centers.

For case-wise routing, lower-dimensional or more strongly regularized routers, richer uncertainty representations such as ensemble-based signals, and additional backbones including self-configuring and transformer-based networks [7,9] may still be investigated. However, the present results indicate that developing a more reliable failure-detection and deferral mechanism is a more fundamental requirement than further optimization of the current heuristic referral score. All proposed approaches will require independent external validation and, ultimately, prospective reader studies.

## 6. Limitations

This study was designed as an exploratory methodological evaluation, not as a clinical validation, and several limitations apply. (i) Only four source datasets (centers) were available, and there was no independent fifth clinical center for external validation. (ii) A single segmentation backbone (a 3D U-Net) was used; results may differ for self-configuring or transformer-based networks. (iii) Each fold used only five labeled target cases and ten target-validation cases per center, so each router was calibrated on 40 cases with 17 features, a high dimensionality relative to the calibration sample. A validation-only simplification check did not show a measurable advantage of the 17-feature representation over reduced alternatives, but the calibration sample is too small for that negative result to establish equivalence or to justify a definitive router redesign. (iv) Five complete repetitions improve the assessment of repeatability but do not replace independent external validation; five seeds provide only a coarse estimate of between-run variability, and the 400 seed-specific observations are not 400 unique patients. (v) There was no prospective deployment, no radiologist reader study, and no workflow evaluation. (vi) Expert reference annotations were used without independent annotation adjudication. (vii) HD95 is reported in voxels. A spacing audit found a stored spacing triplet for every seed-42 test case, but the frozen records did not preserve sufficient axis-order/provenance information to certify a physical-space recomputation from the saved predictions; we therefore did not report unsupported millimeter values. HD95 remains a secondary technical metric and has limited clinical interpretability. (viii) No clinically established acceptable Dice threshold exists for this task. The retrospective Dice ≥0.70 analysis is illustrative only and must not be interpreted as a clinically validated acceptance threshold. (ix) The referral score is a heuristic that was not consistently aligned with segmentation failure, and its threshold transferred poorly from validation to test. (x) AdaSwitch3D requires dual-model inference. (xi) Residual scanner and protocol heterogeneity within and between collections may affect results in ways we could not isolate. (xii) The oracle selectors are upper-bound analyses that require the reference annotation and are not deployable. (xiii) Center-wise and qualitative analyses were performed for seed 42 only, while referral-capture summaries across seeds are descriptive stability analyses rather than confirmatory inferential tests. Pseudo-label confirmation bias in the teacher–student branch also remains possible.

## 7. Conclusions

This study demonstrates both the potential and the limitations of low-resource cross-center adaptation for breast DCE-MRI tumor segmentation. Across five complete repetitions, adaptation improved average performance relative to source-only transfer, but the incremental benefit of AdaSwitch3D routing over teacher–student adaptation was small and seed-dependent, while requiring approximately twice the single-model inference cost. A simpler validation-selected per-center strategy achieved a similar mean Dice.

The principal contribution of this study is therefore methodological and includes an important negative result. The evaluated disagreement- and confidence-based referral heuristic did not provide sufficiently reliable failure detection or a favorable workload–capture trade-off. Severe segmentation failures were frequently missed even when the two candidate models agreed and the router was confident, while achieving high failure capture required reviewing nearly all cases. Retrospective analysis further showed that automatic acceptance did not reliably isolate observations with Dice ≥0.70.

These findings indicate that, under limited target supervision, cross-center adaptation, case-wise model selection, and heuristic referral remain sensitive to data partitioning, calibration, and failure modes that cannot be resolved by inter-model disagreement alone. AdaSwitch3D should therefore be interpreted as an illustrative experimental testbed used to expose and characterize these limitations, rather than as the principal contribution, a preferred deployment architecture, or a clinically validated human-in-the-loop system.

Future progress will require stronger absolute segmentation performance and fundamentally different reliability mechanisms that estimate segmentation failure more directly, including trained segmentation-quality or failure-detection models, learning-to-defer approaches, and appropriately validated risk-controlling selective-prediction methods. Inter-model disagreement may remain useful as a supplementary signal but should not serve as the primary basis for determining whether a segmentation can be accepted without review. Independent external validation and prospective expert-reader studies will ultimately be required before clinical deployment can be considered.

## Figures and Tables

**Table 1:** Analysis hierarchy distinguishing detailed case-level inference from multi-seed stability summaries. Within every seed, checkpoints, postprocessing, routing, and referral calibration were frozen before the untouched test set was evaluated.

Analysis	Status	Data	Summary/Inference
Paired case-level Dice comparisons	Case-level inferential analysis	Seed 42; 80 test cases	Wilcoxon signed-rank tests, Holm correction, center-stratified bootstrap CIs
IoU, precision, recall, failure rates, HD95 (voxels), time	Secondary case-level outcomes	Seed 42	Descriptive; exact paired failure tests with Holm correction
Five-seed repeatability of mean Dice	Seed-level stability analysis	5 seeds; 400 seed-specific observations	Descriptive only: mean ± SD, range, direction of paired differences; no seed-level significance tests
Referral stability	Descriptive stability analysis	5 seeds	Descriptive: referral rate, accepted/referred Dice, low-Dice capture sensitivity, review precision
Referral-policy sensitivity, router simplification, and missed-failure characterization	Post hoc sensitivity analyses	Validation data for threshold/router development; locked test outputs for descriptive policy transfer and failure characterization	No test-label tuning; descriptive sensitivity analyses only
Center-wise results, oracle gap, qualitative cases	Exploratory	Seed 42; oracle gap for 5 seeds	Descriptive only

**Table 2:** Dataset and fold composition for the four-fold leave-one-center-out design. Counts are cases for seed 42; the target-center role sizes (5 labeled, 10 validation, 20 test) were identical in all five seeds, while case assignments were redrawn in each seed. In total, 1231 cases were used.

Target	Source Train	Source Val	Labeled	Unlabeled	Target Val	Target Test
DUKE	1026	24	5	146	10	20
ISPY1	1074	24	5	98	10	20
ISPY2	342	24	5	830	10	20
NACT	1155	24	5	17	10	20

**Table 3:** Training hyperparameters for the three segmentation stages. All stages use the same MONAI 3D U-Net backbone and DiceCELoss(sigmoid=True, squared_pred=True), weight decay $1\times 10^{-5}$, patch size 80×128×128, and sliding-window inference with Gaussian weighting and 0.25 overlap. Identical settings were used in all five seeds.

Setting	Source-Only	Five-Shot	Teacher–Student
Initialization	random	source-only ckpt	source-only ckpt
Learning rate	$1\times 10^{-4}$	$1\times 10^{-5}$	$1\times 10^{-5}$
Steps per epoch	250	75	100
Max epochs	80	20	20
Patience	12	5	5
Labeled target cases	—	5	5
Sampling	center-balanced, fg 0.90	target labeled	sup/pseudo 0.50/0.50
Supervised weight	—	—	1.00
Pseudo-label weight	—	—	0.30
Pseudo thresholds	—	—	bin 0.50; fg 0.90; bg 0.10
Selection set	source val	target val	target val

**Table 4:** Referral stability across seeds (80 untouched test cases per seed). The referral threshold was calibrated in each seed to refer 20% of that seed’s validation cases and was not changed afterwards. Low-Dice: AdaSwitch3D Dice <0.20. Capture sensitivity = low-Dice cases referred/all low-Dice cases; review precision = low-Dice cases referred/all referred cases. Accepted and referred Dice are descriptive subset statistics.

Seed	Referral Rate (Referred/80)	Accepted Dice (*n*)	Referred Dice (*n*)	Low-Dice Cases	Low-Dice Referred	Capture Sensitivity	Review Precision
42	35.00% (28)	0.3989 (52)	0.2249 (28)	38	16	42.1%	57.1%
123	56.25% (45)	0.4056 (35)	0.2273 (45)	39	28	71.8%	62.2%
2024	28.75% (23)	0.4712 (57)	0.2605 (23)	32	13	40.6%	56.5%
2025	31.25% (25)	0.4190 (55)	0.2892 (25)	35	15	42.9%	60.0%
2026	23.75% (19)	0.4537 (61)	0.4388 (19)	23	5	21.7%	26.3%
Pooled (400)	35.00% (140)	—	—	167	77	46.1%	55.0%

**Table 5:** Mean test Dice by seed for every strategy (80 untouched test cases per seed; 20 per center). The last column gives the mean ± sample SD across the five seeds. Center-best: for each held-out center, the strategy with the highest target-validation Dice, applied to all test cases of that center. Oracle selectors use the expert reference annotation and are non-deployable upper bounds.

Strategy	Seed 42	Seed 123	Seed 2024	Seed 2025	Seed 2026	Mean ± SD
Source-only	0.2616	0.3338	0.4019	0.3068	0.3205	0.3249±0.0509
Five-shot	0.2962	0.2967	0.3936	0.3772	0.3990	0.3525±0.0518
Teacher–student	0.3231	0.3245	0.4063	0.3757	0.4280	0.3715±0.0474
Center-best (validation)	0.3103	0.3653	0.4099	0.3730	0.4231	0.3763±0.0442
AdaSwitch3D	0.3380	0.3053	0.4106	0.3785	0.4502	0.3765±0.0573
Upper bounds; non-deployable; not part of AdaSwitch3D						
Oracle adapted-model	0.3670	0.3638	0.4671	0.4023	0.4645	0.4130±0.0506
Oracle all-model	0.4031	0.4565	0.5019	0.4393	0.5068	0.4615±0.0436

**Table 6:** Per-seed paired differences in mean test Dice, AdaSwitch3D minus each comparator. “Positive” is the number of seeds in which AdaSwitch3D was higher. Descriptive seed-level summaries; no significance tests were applied across seeds.

AdaSwitch3D Minus	42	123	2024	2025	2026	Mean (SD)	Positive
Source-only	+0.0764	−0.0284	+0.0087	+0.0717	+0.1296	+0.0516 (0.0620)	4/5
Five-shot	+0.0418	+0.0087	+0.0171	+0.0013	+0.0511	+0.0240 (0.0215)	5/5
Teacher–student	+0.0149	−0.0192	+0.0044	+0.0027	+0.0221	+0.0050 (0.0157)	4/5
Center-best	+0.0277	−0.0600	+0.0007	+0.0055	+0.0271	+0.0002 (0.0358)	4/5

**Table 7:** Overall performance on the untouched seed-42 test set (n=80; 20 cases per center). Values are reported as means unless otherwise specified. Dice 95% confidence intervals were estimated from 5000 center-stratified bootstrap resamples. HD95 is reported as the median finite value in voxels and is included only as a secondary technical boundary metric, not as a physical distance (see Section 3, Metrics). Oracle selectors are non-deployable upper bounds.

Strategy	Dice	95% CI	Median	IoU	Prec.	Rec.	Fail %	HD95 (vox)	Time (s)
Source-only	0.262	0.207–0.317	0.167	0.188	0.295	0.437	32.50	52.145	0.090
Five-shot	0.296	0.237–0.358	0.223	0.210	0.345	0.546	22.50	46.311	0.081
Teacher–student	0.323	0.255–0.392	0.197	0.244	0.366	0.485	31.25	23.246	0.081
Validation-best (center)	0.310	—	0.217	0.223	0.372	0.557	21.25	43.020	0.081
AdaSwitch3D	0.338	0.273–0.403	0.245	0.253	0.419	0.521	23.75	26.840	0.164
Upper bounds; non-deployable; not part of AdaSwitch3D									
Oracle adapted-model selector	0.367	0.303–0.434	0.322	0.275	0.434	0.553	20.00	27.893	0.163
Oracle all-model selector	0.403	—	0.419	0.307	0.468	0.567	16.25	21.932	0.253

**Table 8:** Center-wise mean test Dice by strategy for seed 42 (20 cases per center). Performance varied across centers, and no strategy consistently achieved the highest Dice across all four centers.

Center	Source-Only	Five-Shot	Teacher–Student	Val-Best	AdaSwitch3D
DUKE	0.137	0.302	0.326	0.326	0.326
ISPY1	0.216	0.228	0.260	0.260	0.235
ISPY2	0.196	0.286	0.229	0.286	0.300
NACT	0.497	0.369	0.478	0.369	0.491

**Table 9:** Paired Dice comparisons for seed 42 (two-sided Wilcoxon signed-rank tests) with raw and Holm-adjusted *p*-values. Differences are mean paired Dice differences; 95% CIs are from 5000 center-stratified bootstrap resamples. Significant comparisons after correction are in bold.

Comparison	Δ Dice	95% CI	Raw *p*	Holm *p*
Five-shot vs. source-only	+0.035	−0.010 to 0.078	0.254	0.508
Teacher–student vs. source-only	+0.061	0.005 to 0.117	0.012	0.060
**AdaSwitch3D vs. source-only**	+0.076	0.024 to 0.127	**0.003**	**0.021**
Teacher–student vs. five-shot	+0.027	−0.015 to 0.066	0.079	0.274
**AdaSwitch3D vs five-shot**	+0.042	0.012 to 0.071	**0.003**	**0.021**
AdaSwitch3D vs. teacher–student	+0.015	−0.010 to 0.043	0.255	0.508
AdaSwitch3D vs. validation-best	+0.028	−0.003 to 0.057	0.068	0.274

**Table 10:** AdaSwitch3D routing and referral analysis for seed 42 on validation and on the untouched test set. Accepted- and referred-case Dice are descriptive statistics for those subsets only.

Quantity	Validation (40)	Test (80)
Referral rate	20.0%	35.0% (28/80)
Accepted mean Dice	0.490	0.399 (*n* = 52)
Referred mean Dice	0.347	0.225 (*n* = 28)
Routing accuracy	67.5%	—
Low-Dice cases (<0.20)	—	38
Low-Dice cases referred	—	16
Low-Dice capture sensitivity	—	42.1%
Review precision (low-Dice)	—	57.1%
Teacher–student selected	—	53
Five-shot selected	—	27
Mean router confidence	—	0.751
Mean model-agreement Dice	—	0.560

## Data Availability

The imaging data are publicly available through The Cancer Imaging Archive as the DUKE, ISPY1, ISPY2, and NACT breast DCE-MRI collections and are aggregated with expert segmentations in the MAMA-MIA dataset [3]. The supplementary tables and figures accompany this article. Additional research artifacts, including split manifests, frozen configuration files, case-level results, and the five-seed summary and sensitivity-analysis data, are available from the corresponding author upon request. The training, evaluation, adaptation, preprocessing, and AdaSwitch3D routing scripts, together with the requirements file specifying the software environment, are available from the corresponding author upon reasonable request.

## Supplementary Materials

The following supporting information can be downloaded at https://www.mdpi.com/article/10.3390/diagnostics16203378/s1. Table S1: Frozen validation-calibrated probability thresholds by held-out center and method; Table S2: Paired failure-rate comparisons with raw and Holm-adjusted *p*-values; Table S3: Frozen referral-threshold transfer analysis; Table S4: Five-seed summary of mean test Dice, paired differences, and referral statistics; Table S5: Five-seed referral-policy sensitivity; Table S6: Validation-only router-complexity sensitivity across five seeds; Table S7: Feature comparison for missed versus referred low-Dice observations; Table S8: Seed-42 physical-spacing audit for HD95; Table S9: Retrospective high-Dice case analysis across the five locked test evaluations; Figure S1: Worst case per center (2D) by frozen AdaSwitch3D Dice; Figure S2: 3D surface renderings for representative best, median, and worst cases per center; Figure S3: Failure rates on the untouched test set by strategy; Figure S4: Median finite HD95 (voxels) by strategy; Figure S5: Oracle gap by seed; Figure S6: Validation-only referral-policy sensitivity; Figure S7: Median feature comparison for missed versus referred low-Dice observations.
