# Supplementary material for: AdaSwitch3D: Reliability-Aware Human-in-the-Loop Artificial Intelligence for Breast Tumor Segmentation in Dynamic Contrast-Enhanced MRI Across Unseen Centers
Source: Diagnostics (Basel). 2026 Oct 17;16(20):3378. doi: 10.3390/diagnostics16203378 (PMC13703028; doi:10.3390/diagnostics16203378)
Supplement: Supplementary file 1 [file diagnostics-16-03378-s001.zip › diagnostics-4507664-supplementary.pdf]

diagnostics

MDPI

Article

Supplementary Materials: AdaSwitch3D: Reliability-Aware Human-in-the-Loop Artificial Intelligence for Breast Tumor Segmentation in Dynamic Contrast-Enhanced MRI Across Unseen Centers

Easa Alalwany [1,2,*] and Bader Alsharif [3]

[1] Department of Computer Science, College of Computer Science and Engineering, Taibah University, Yanbu 46421, Saudi Arabia
[2] Health and Life Research Center, Taibah University, Madinah 42353 , Saudi Arabia
[3] Department of Electrical Engineering and Computer Science, Florida Atlantic University, Boca Raton, FL 33431, USA; balsharif2020@fau.edu
* Correspondence: ealwani@taibahu.edu.sa

1. Frozen validation-calibrated probability thresholds, seed 42 (Table S1)

Table S1 lists the per-fold, per-method probability thresholds selected on the ten disjoint target-validation cases in each fold. These thresholds were frozen before the one-time untouched-test evaluation. Connected-component filtering was additionally applied: the largest predicted component was retained and smaller isolated components below the frozen validation-calibrated minimum-volume setting were removed. No morphological hole filling was applied.

Table S1. Frozen validation-calibrated probability thresholds by held-out center and method.

Held-out center	Source-only	Five-shot	Teacher–student
DUKE	0.60	0.25	0.25
ISPY1	0.55	0.65	0.75
ISPY2	0.20	0.40	0.30
NACT	0.50	0.25	0.45

2. Failure-rate statistics, seed 42 (Table S2)

Table S2 reports paired failure-rate comparisons for two failure definitions: complete failure (Dice $= 0$) and Dice < 0.20. Comparisons used exact paired tests on discordant case pairs with Holm correction within each definition. **No failure-rate comparison remained significant after correction** at $\alpha = 0.05$.

Check for updates

Received: 2 August 2026
Revised: 11 October 2026
Accepted: 14 October 2026
Published: 17 October 2026

Copyright: © 2026 by the authors. Licensee MDPI, Basel, Switzerland. This article is an open access article distributed under the terms and conditions of the Creative Commons Attribution (CC BY) license.

Table S2. Paired failure-rate comparisons with raw and Holm-adjusted p-values. "Impr." and "Wors." are the numbers of discordant cases favoring the comparison and reference method, respectively.

Definition	Comparison	Ref %	Comp %	Impr.	Wors.	Holm p
Complete failure	Five-shot vs source-only	32.5	22.5	11	3	0.402
Complete failure	Teacher–student vs source-only	32.5	31.25	8	7	1.000
Complete failure	AdaSwitch3D vs source-only	32.5	23.75	11	4	0.474
Complete failure	Teacher–student vs five-shot	22.5	31.25	2	9	0.402
Complete failure	AdaSwitch3D vs five-shot	22.5	23.75	1	2	1.000
Complete failure	AdaSwitch3D vs teacher–student	31.25	23.75	7	1	0.402
Complete failure	AdaSwitch3D vs validation-best	21.25	23.75	1	3	1.000
Dice $<$ 0.20	Five-shot vs source-only	55.0	48.75	10	5	1.000
Dice $<$ 0.20	Teacher–student vs source-only	55.0	51.25	11	8	1.000
Dice $<$ 0.20	AdaSwitch3D vs source-only	55.0	47.5	12	6	1.000
Dice $<$ 0.20	Teacher–student vs five-shot	48.75	51.25	4	6	1.000
Dice $<$ 0.20	AdaSwitch3D vs five-shot	48.75	47.5	3	2	1.000
Dice $<$ 0.20	AdaSwitch3D vs teacher–student	51.25	47.5	4	1	1.000
Dice $<$ 0.20	AdaSwitch3D vs validation-best	50.0	47.5	4	2	1.000

3. Referral-threshold transfer analysis, seed 42 (Table S3)

Table S3 summarizes how the validation-frozen referral threshold transferred from the target-validation set to the untouched target-test set. The threshold was selected only on the 40 target-validation cases to produce a 20% referral rate and was then applied without adjustment to the 80 untouched target-test cases. This analysis does not retune the threshold and does not change the final AdaSwitch3D results. Instead, it describes whether the frozen referral operating point remained stable when applied to unseen test cases.

The threshold $\tau = 0.3369297453305864$ referred 8 of 40 validation cases, matching the intended 20% validation referral rate. On the untouched test set, the same threshold referred 28 of 80 cases, corresponding to a higher referral rate of 35.0%. Among the 38 test cases with Dice < 0.20, 16 were referred, giving a low-Dice capture sensitivity of 42.1%. Among the 28 referred test cases, 16 had Dice < 0.20, giving a review precision of 57.1%. These findings show that the reliability score enriched the review queue for poor cases but did not capture most low-Dice cases. Therefore, the referral mechanism should be interpreted as a review-prioritization signal rather than a safety mechanism. The five-seed repetition (main text, Section 4.2, and Table S4 below) showed that this seed-42 behavior was not consistently reproduced.

https://doi.org/10.3390/diagnostics16203378

Table S3. Frozen referral-threshold transfer analysis. The referral threshold was selected on the target-validation set to produce a 20% referral rate and was then applied without adjustment to the untouched target-test set. This table reports the primary frozen operating point; a separate validation-only multi-threshold sensitivity analysis is reported in Table S5 and Figure S6.

Metric	Target validation	Untouched test
Cases	40	80
Threshold τ	0.3369	0.3369
Referral rate	20.0%	35.0%
Referred cases	8/40	28/80
Accepted mean Dice	0.490	0.399
Referred mean Dice	0.347	0.225
Low-Dice cases, Dice $<$ 0.20	N/R	38
Low-Dice cases referred	N/R	16
Low-Dice capture sensitivity	N/R	42.1%
Review precision for low-Dice cases	N/R	57.1%

N/R = not reported for that split. Low-Dice was defined as Dice $<$ 0.20. Accepted and referred Dice values are descriptive statistics for those subsets only and do not represent performance across all cases.

4. Worst cases per center (Figure S1)

Figure S1 shows the deterministically selected worst case (by frozen AdaSwitch3D Dice) for each center. All four worst cases had Dice $=$ 0.000 and were flagged for referral by the reliability score.

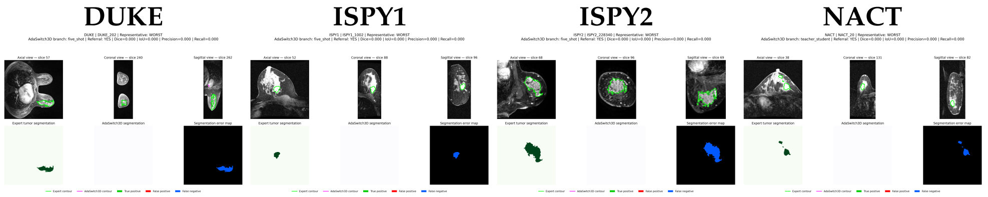

Figure S1. Worst case per center (2D) by frozen AdaSwitch3D Dice; all had Dice $=$ 0.000 and were referred for review.

5. 3D renderings (Figure S2)

Figure S2 shows 3D surface renderings for the representative best, median, and worst cases per center. Each corresponds to the matching 2D panel with `_2D.png` replaced by `_3D.png`.

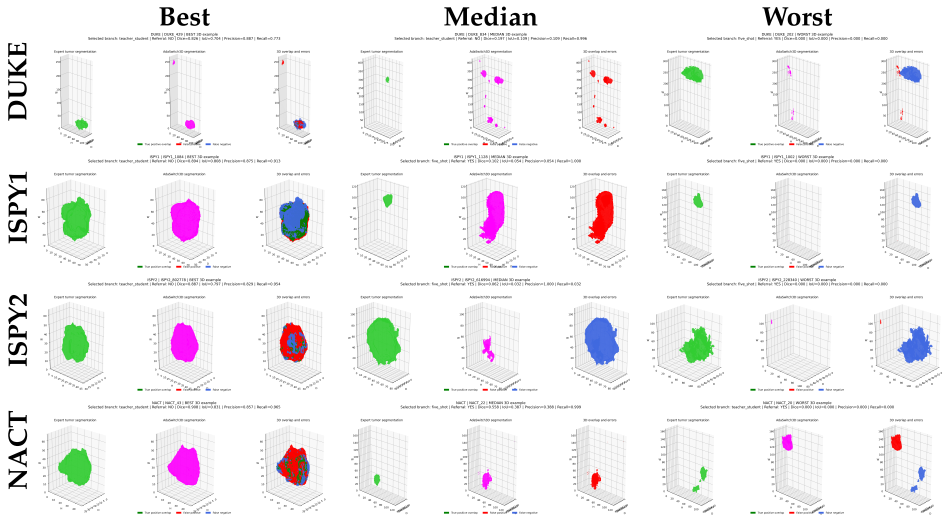

Figure S2. 3D surface renderings for representative best, median, and worst cases per center (rows: DUKE, ISPY1, ISPY2, NACT).

6. Failure-rate comparison by strategy (Figure S3)

Figure S3 compares the two failure definitions across the deployable strategies on the untouched test set. Five-shot fine-tuning had the lowest complete-failure rate and AdaSwitch3D the lowest Dice < 0.20 rate, but no failure-rate comparison remained significant after Holm correction (Table S2).

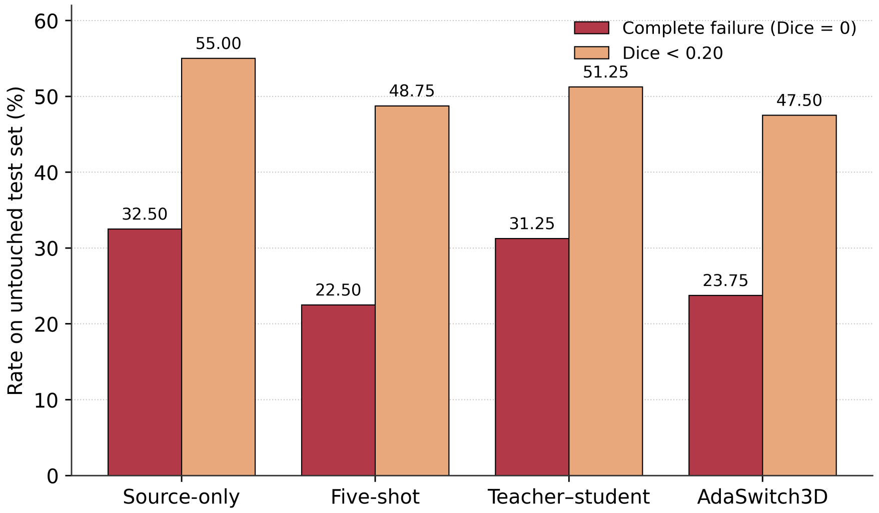

Figure S3. Failure rates on the untouched test set ($n = 80$) by strategy, under two definitions: complete failure (Dice = 0) and Dice < 0.20. Complete failures persisted under every strategy; no pairwise failure-rate difference was significant after Holm correction.

7. Median finite HD95 by strategy (Figure S4)

Figure S4 shows the median finite HD95 in voxels for each strategy. A dedicated audit of the 80 seed-42 test records found one stored spacing candidate for every case, but the frozen records did not preserve the axis-order/provenance needed to map that triplet unambiguously to the prediction-array axes or to certify consistency with the documented preprocessing grid. We therefore did not convert the stored voxel HD95 values to millimeters. Because physical surface distances on an anisotropic grid depend on direction, multiplying the voxel HD95 by a single constant would be invalid. These values support only relative technical comparison and are not physical distances. Finite values were sum-

https://doi.org/10.3390/diagnostics16203378

marized separately because empty-mask cases can yield infinite or undefined distances; the spacing audit is summarized in Table S8.

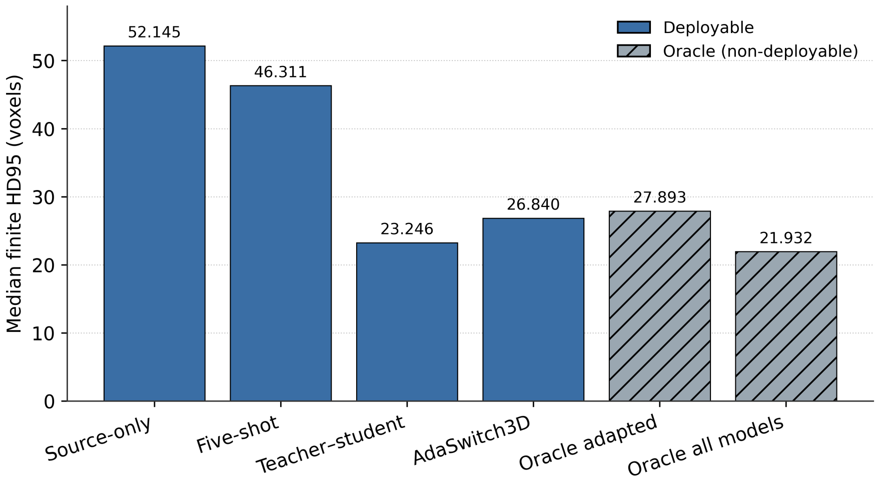

Figure S4. Median finite 95th-percentile Hausdorff distance (HD95, in voxels) by strategy on the untouched test set. Lower is better. Oracle strategies are upper bounds and are non-deployable, since they require the expert reference annotation to select a branch.

8. Five-seed summary (Table S4)

Table S4 collects the five-seed values reported in the main text (Tables 4–6). Seeds 42, 123, 2024, 2025, and 2026 each denote a complete, independent repetition of the pipeline (new partition and five-shot selection, retraining, adaptation, router and referral recalibration, and one-time evaluation on 80 untouched test cases). Summaries are descriptive; no significance tests were applied across seeds. A machine-readable version is provided in the accompanying spreadsheet.

Table S4. Five-seed summary. Upper block: mean test Dice. Middle block: AdaSwitch3D minus comparator. Lower block: referral statistics (threshold calibrated to 20% referral on each seed's validation set). SD is the sample standard deviation across seeds.

	**42**	**123**	**2024**	**2025**	**2026**	**Mean (SD)**
Source-only	0.2616	0.3338	0.4019	0.3068	0.3205	0.3249 (0.0509)
Five-shot	0.2962	0.2967	0.3936	0.3772	0.3990	0.3525 (0.0518)
Teacher–student	0.3231	0.3245	0.4063	0.3757	0.4280	0.3715 (0.0474)
Center-best	0.3103	0.3653	0.4099	0.3730	0.4231	0.3763 (0.0442)
AdaSwitch3D	0.3380	0.3053	0.4106	0.3785	0.4502	0.3765 (0.0573)
Oracle adapted-model	0.3670	0.3638	0.4671	0.4023	0.4645	0.4130 (0.0506)
Oracle all-model	0.4031	0.4565	0.5019	0.4393	0.5068	0.4615 (0.0436)
Ada − source-only	+0.0764	−0.0284	+0.0087	+0.0717	+0.1296	+0.0516 (0.0620)
Ada − five-shot	+0.0418	+0.0087	+0.0171	+0.0013	+0.0511	+0.0240 (0.0215)
Ada − teacher–student	+0.0149	−0.0192	+0.0044	+0.0027	+0.0221	+0.0050 (0.0157)
Ada − center-best	+0.0277	−0.0600	+0.0007	+0.0055	+0.0271	+0.0002 (0.0358)
Oracle adapted − Ada	+0.0290	+0.0585	+0.0565	+0.0238	+0.0144	+0.0364 (0.0199)
Referral rate (%)	35.00	56.25	28.75	31.25	23.75	35.00 (12.56)
Accepted mean Dice	0.3989	0.4056	0.4712	0.4190	0.4537	—
Referred mean Dice	0.2249	0.2273	0.2605	0.2892	0.4388	—
Low-Dice cases / referred	38 / 16	39 / 28	32 / 13	35 / 15	23 / 5	167 / 77 (pooled)
Capture sensitivity (%)	42.1	71.8	40.6	42.9	21.7	43.8 (17.9)
Review precision (%)	57.1	62.2	56.5	60.0	26.3	52.4 (14.8)

9. Oracle gap by seed (Figure S5)

Figure S5 shows teacher–student adaptation, AdaSwitch3D, and the two non-deployable oracle selectors for each seed. The gap between AdaSwitch3D and the oracle adapted-model selector (0.0144–0.0585) indicates that label-free routing recovered only part of the available case-level selection benefit.

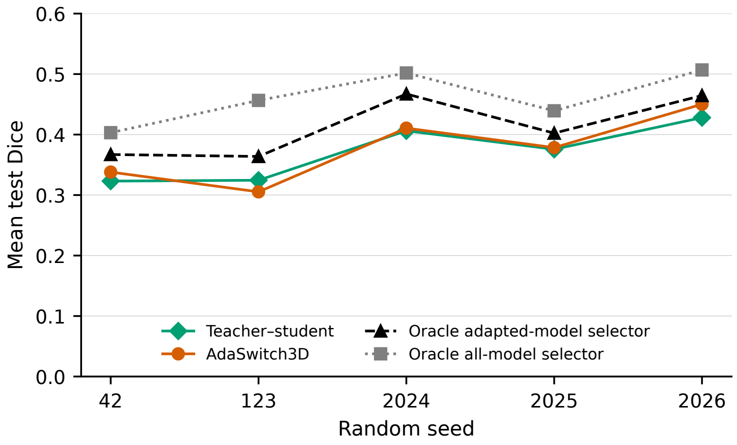

Figure S5. Oracle gap by seed. Mean test Dice for teacher–student adaptation, AdaSwitch3D, and the non-deployable oracle adapted-model and all-model selectors (80 test cases per seed). The y-axis starts at zero.

10. Validation-only referral-policy sensitivity (Table S5 and Figure S6)

The primary analysis used the frozen threshold calibrated to a 20% validation referral burden in each seed. As a post hoc sensitivity analysis, alternative thresholds were selected from validation data only and then applied unchanged to the already-locked test scores. No test label was used to select or modify a threshold. Review-burden targets of 10%, 30%, 40%, and 50% and validation low-Dice capture targets of 50%, 70%, and 90% were evaluated. Center-specific thresholds were not estimated because only ten validation cases were available per center per seed.

Table S5. Five-seed referral-policy sensitivity. Values are mean (sample SD) across seeds. Test review burden is the fraction referred on the untouched test set; low-Dice capture sensitivity is the fraction of Dice < 0.20 observations referred; review precision is the fraction of referred observations with Dice < 0.20. All operating points were selected from validation data only.

Validation policy	Test burden	Accepted Dice	Failure capture	Review precision
10% burden	0.238 (0.087)	0.415 (0.047)	0.317 (0.160)	0.540 (0.191)
30% burden	0.398 (0.139)	0.426 (0.038)	0.478 (0.181)	0.505 (0.146)
40% burden	0.480 (0.106)	0.430 (0.050)	0.580 (0.101)	0.508 (0.100)
50% burden	0.568 (0.088)	0.412 (0.061)	0.629 (0.096)	0.462 (0.087)
50% validation failure capture	0.525 (0.193)	0.409 (0.041)	0.587 (0.143)	0.490 (0.130)
70% validation failure capture	0.718 (0.177)	0.317 (0.146)	0.718 (0.135)	0.434 (0.136)
90% validation failure capture	0.960 (0.047)	0.198 (0.265)	0.969 (0.033)	0.424 (0.090)

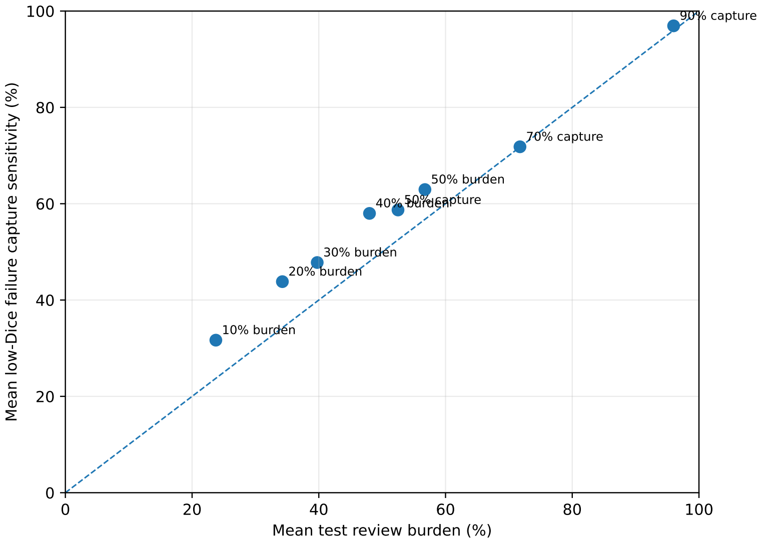

Figure S6. Validation-only referral-policy sensitivity. Each point shows the mean test review burden and mean low-Dice failure-capture sensitivity across the five seeds for an operating point selected from validation data only. The diagonal is shown only as a visual reference. Higher capture required substantially higher review burden.

11. Validation-only router-complexity sensitivity (Table S6)

The original router uses 17 label-free features with class-balanced logistic regression. A validation-only sensitivity check compared that representation with predefined reduced 5-feature and 3-feature variants and stronger L_2 regularization. No test label was used for feature selection, regularization, or router replacement; the original frozen router remained the model evaluated on the untouched test sets. In this small validation-only analysis, the out-of-fold Dice summary was indistinguishable across the tested configurations, providing no evidence that the full 17-feature representation improved validation performance over the reduced alternatives.

Table S6. Validation-only router-complexity sensitivity across five seeds. OOF = out-of-fold. The identical summaries should be interpreted as a negative sensitivity result in a very small calibration sample, not as evidence of equivalence.

Router	Features	C	Mean OOF Dice (SD)
Full	17	1.00	0.4284 (0.0426)
Full, stronger regularization	17	0.10	0.4284 (0.0426)
Full, strongest tested regularization	17	0.01	0.4284 (0.0426)
Reduced	5	1.00	0.4284 (0.0426)
Reduced, stronger regularization	5	0.10	0.4284 (0.0426)
Reduced	3	1.00	0.4284 (0.0426)
Reduced, stronger regularization	3	0.10	0.4284 (0.0426)

12. Missed low-Dice failure characterization (Table S7 and Figure S7)

Across the five locked test evaluations there were 167 AdaSwitch3D observations with Dice < 0.20. Of these, 77 were referred and 90 were accepted automatically. We compared already-computed label-free router features between the missed and referred low-Dice groups. This analysis is descriptive and did not influence any threshold or prediction.

Table S7. Feature comparison for missed versus referred low-Dice observations across the five locked test evaluations. Values are medians unless otherwise indicated.

Feature	Missed low-Dice ($n = 90$)	Referred low-Dice ($n = 77$)
Router confidence	0.846	0.655
Inter-model agreement Dice	0.840	0.162
Mean absolute probability difference	0.115	0.179
Log predicted-volume difference	0.217	0.907
Foreground-confidence difference	0.099	0.111
Entropy difference	0.053	0.068
Both candidate masks empty, count	20	—

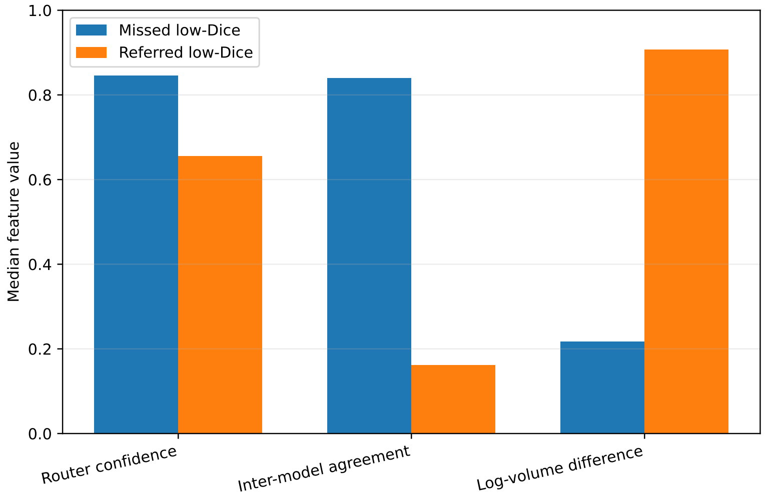

Figure S7. Median router confidence, inter-model agreement, and log predicted-volume difference for missed versus referred low-Dice observations. Missed failures frequently occurred when the two candidate models agreed and the router was confident, illustrating a limitation of disagreement-based referral.

13. Physical-spacing audit for HD95 (Table S8)

The frozen seed-42 test records were audited for spacing metadata before attempting a millimeter HD95 recomputation. Each of the 80 records contained one spacing candidate, but the serialized field did not identify the axis ordering/provenance required to map its components unambiguously to the model prediction array. The stored triplet was $(1.0, 1.0, 1.5)$ for the audited records, whereas the documented preprocessing target grid is anisotropic and defined separately in the main Methods. Because a physically correct Hausdorff distance requires distances to be computed in a coordinate system with verified axis-specific spacing, we did not multiply voxel HD95 by any constant or report an uncertified millimeter value.

Table S8. Seed-42 spacing audit used to assess whether stored voxel HD95 could be converted defensibly to millimeters.

Audit item	Result
Untouched test cases audited	80
Cases with a spacing candidate	80/80
Candidates per case	1
Stored spacing triplet	$(1.0, 1.0, 1.5)$
Axis-order/provenance relative to prediction array	Not preserved in frozen record
Certified millimeter HD95 reconstruction	Not possible from frozen artifacts alone
Reported HD95 unit	Voxels (secondary technical metric)

14. Retrospective high-Dice case analysis (Table S9)

Table S9 provides a post hoc descriptive analysis of how frequently AdaSwitch3D achieved relatively high segmentation overlap and whether those observations were retained by the frozen referral rule. Dice thresholds of 0.50, 0.60, 0.70, and 0.80 were evaluated retrospectively across the 400 seed-specific untouched-test observations. These thresholds were not used to tune the router, referral score, or any prediction. In particular, Dice ≥ 0.70 is used only as an illustrative high-quality threshold motivated by the reviewer comment and should not be interpreted as a clinically validated cutoff.

The analysis distinguishes two quantities. "Good-case acceptance" is the fraction of observations meeting a given Dice threshold that were not referred for review. "Accepted-case precision" is the fraction of all automatically accepted observations that actually met that Dice threshold. Although 79.4% of observations with Dice $\geq$ 0.70 were accepted, only 29.6% of all accepted observations achieved Dice $\geq$ 0.70. Thus, the current referral mechanism retained many high-Dice observations but did not reliably isolate them from lower-quality segmentations. Because true Dice requires a reference segmentation and is unavailable prospectively, these results are descriptive only and do not support automatic acceptance at deployment.

Table S9. Retrospective high-Dice case analysis across the five locked test evaluations ($n = 400$ seed-specific observations). "Good-case acceptance" is the fraction of observations meeting the Dice threshold that were automatically accepted. "Accepted-case precision" is the fraction of all accepted observations that met the Dice threshold. Thresholds were evaluated post hoc and were not used for model selection, calibration, or referral decisions.

Dice threshold	Cases meeting threshold	Fraction of all cases	Good cases accepted	Good-case acceptance	Accepted-case precision
$\geq$ 0.50	173/400	43.3%	135/173	78.0%	51.9%
$\geq$ 0.60	140/400	35.0%	113/140	80.7%	43.5%
$\geq$ 0.70	97/400	24.3%	77/97	79.4%	29.6%
$\geq$ 0.80	44/400	11.0%	33/44	75.0%	12.7%

For the illustrative Dice $\geq$ 0.70 threshold, the fraction of high-Dice observations across the five seeds was 0.242 ± 0.041, the good-case acceptance rate was 0.784 ± 0.092, and accepted-case precision was 0.296 ± 0.051 (mean $\pm$ sample SD). Center-level accepted-case precision was 0.194 for DUKE, 0.229 for ISPY1, 0.385 for ISPY2, and 0.394 for NACT, further demonstrating that the current referral rule cannot be interpreted as a reliable prospective quality estimator.

https://doi.org/10.3390/diagnostics16203378
